# Chromium-free Cu@Mg/γ-Al_2_O_3_ – an active catalyst for selective hydrogenation of furfural to furfuryl alcohol†

**DOI:** 10.1039/d0ra08754h

**Published:** 2020-11-10

**Authors:** Racha Arundhathi, Panyala Linga Reddy, Chanchal Samanta, Bharat L. Newalkar

**Affiliations:** Corporate Research & Development Centre, Bharat Petroleum Corporation Limited Greater Noida Uttar Pradesh-201306 India newalkarbl@bharatpetroleum.in; Department of Chemistry, Indian Institute of Technology Bombay Powai Mumbai-400076 India

## Abstract

Development of a chromium (Cr)-free hydrogenation catalyst is very important to replace the existing hazardous Cr based catalyst used in the furfural hydrogenation to furfuryl alcohol. Herein, we report synthesis of well-dispersed copper nanoparticles supported on hydrothermally stable magnesium doped alumina (Cu@Mg/γ-Al_2_O_3_) for selective hydrogenation of furfural to furfuryl alcohol. The prepared catalyst was characterized by X-ray Photoelectron Spectroscopy (XPS), Auger Electron Spectroscopy (AES), Powder X-ray Diffraction (PXRD), Surface Area Analysis (SAA), High Resolution-Transmission Electron Microscopy (HR-TEM), Temperature Programmed Reduction/Desorption (TPR/TPD) and Inductively Coupled Plasma-Atomic Emission Spectroscopy (ICP-AES) to understand textural properties of the catalyst. The prepared catalyst was found to be highly active and selective with 99% conversion of furfural and 94% selectivity for furfuryl alcohol under solvent free conditions at 443.15 K and 2 MPa of hydrogen pressure. It was also observed that the Cu@Mg/γ-Al_2_O_3_ catalyst is reusable (up to six runs) while maintaining its high activity and selectivity (≥94%) in the hydrogenation of furfural to furfuryl alcohol.

## Abstract 2

A catalyst for selective hydrogenation of furfural to furfural alcohol in a solvent free environment.

## Graphical abstract

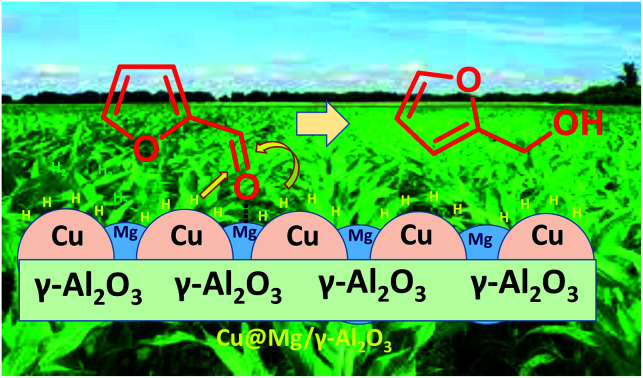

## 1. Introduction

Furfural (FF) is considered as one of the most promising value-added chemicals that can be produced from lignocellulosic biomass and it is a useful raw material for C_4_ and C_5_ based chemical production.^1,2^ Furfural is industrially produced through the acid hydrolysis of agro-based biomass. Alternatively, it can be produced from woody biomass in an integrated forest biorefinery. Furfural is an important platform molecule and can be converted to other useful chemicals. Thus, many efforts have been devoted to the development of catalytic transformations of FF to other value-added chemicals such as furfuryl alcohol (FA), tetrahydro furfuryl alcohol (THFA) cyclopenatnone, 2-methyl furan (2-MF), 1,2 pentane diols, tetrahydrofuran (THF).^3^ Furfuryl alcohol (FA) is one of the most important derivative of furfural which has much wider applications than furfural. Around 62% of the globally produced furfural is converted to FA.^4^ FA is extensively used as a monomer for cross-linked polymers to produce furan resins, which are used in foundry binders. These resins have excellent chemical, thermal and mechanical properties along with the capability of resisting corrosion.^5^

Commercially, FA is produced through catalytic hydrogenation of furfural using Cu–Cr based catalyst. Although Cu–Cr based catalyst is active and highly selective for furfural hydrogenation, negative environmental effect of Cr is a major concern for this catalyst. Despite the high yield associated with the traditional Cu–Cr catalyst, it is not ideal due to the presence of Cr_2_O_3_ (chromium oxide) which can cause severe environmental pollution.^6^ Therefore, development of Cr-free catalytic system with high activity, selectivity, recyclability and able to perform reaction under industrially relevant conditions is highly desirable for hydrogenation of FF to FA. Thus, industries are looking for Cr-free catalyst with similar performance in terms of selectivity and activity. In recent years, various Cr-free catalytic systems based on Pt, Pd, Ru, Rh, Ir and Au have been reported for hydrogenation of furfural and have shown good catalytic conversation under mild reaction conditions.^7^ However, undesired product formation because of high catalytic activity and cost factors of precious metals have limited the commercial applications of noble metal based catalysts. In comparison to this, development of non-precious catalyst system has attractive advantages to meet the challenges of cost effectiveness and sustainability for industrial usage. In search of this, supported and non-supported metal heterogeneous catalysts based on Fe, Co, Ni and Cu were explored.^8^ Along with attractive advantages, these catalysts have some disadvantages like low activity, need of higher reaction temperature and lower selectivity towards the desired products. Therefore, to enhance catalytic activity and selectivity, doping small amount of appropriate heteroatom have been explored.

Magnesium supported materials found to exert high catalytic activity in the furfural hydrogenation.^9^ Various Mg incorporated catalysts such as Cu–Mg/ZnO,^10,11^ Mg-doped Pt/Al_2_O_3_,^12^ Ni–Mg/SiO_2_,^13^ Mg-doped Co–Ni nanocatalyst,^14^ Mg–TiO_2_,^15^ Mg doped ZnO,^16^ graphene oxide/WS_2_/Mg-doped ZnO nanocomposite^17^ have been explored in hydrogenation, hydrogen storage and other type reactions.^10–17^ However, limited detailed studies are reported in the literature to demonstrate the performance of magnesium doped catalyst in FF hydrogenation to FA. Therefore, we have synthesized and studied performance of magnesium doped heterogeneous Cu@Mg/γ-Al_2_O_3_ catalyst for the selective hydrogenation of FF to FA under solvent free conditions.

## 2. Experimental

### 2.1 Materials and methods

All reagents were purchased from commercial suppliers and used without further purification. All hydrogenation experiments were carried out under hydrogen. Column chromatography was carried out with Merck silica gel 60–120 mesh and the products were characterized and quantified by GC detection. ^1^H NMR and ^13^C NMR (300 or 400 MHz and 75 or 100 MHz, respectively) spectra were recorded in CDCl_3_. Chemical shifts (*δ*) are reported in ppm using TMS as an internal standard, and spin–spin coupling constants (*J*) are given in Hz.

### 2.2 Catalyst preparation

Cu@Mg/γ-Al_2_O_3_ catalyst was prepared in three steps as follows.

Step 1: 200 mL of deionised water was taken in a 1 L four neck round bottom flask and equipped with an overhead mechanical stirrer. Mg(NO_3_)_2_ ·6H_2_O (6.63 g, 0.025 moles) was dissolved and 45 grams of γ-Al_2_O_3_ (45 g, 0.4413 moles) was added to Mg(NO_3_)_2_·6H_2_O dissolved solution. The resulting slurry was kept for stirring for 2 h and aged at 70 °C for 4 h. The solid product was isolated by filtration and dried at 110 °C for 12 h in an air oven. Mg doped alumina catalyst was then calcined at 350 °C in the presence of air for 5 h to obtain MgO/γ-Al_2_O_3_ and then cooled to room temperature.

Step 2: in a 250 mL round bottomed flask 100 mL of deionized water was taken and 22.11 g of Cu(NO_3_)_2_·3H_2_O (0.0915 moles) was added under stirring conditions at room temperature for complete dissolution of copper salt. To this solution, 20 g of calcined MgO/γ-Al_2_O_3_ catalyst added and stirred at room temperature for 2 h. The pH of the reaction mixture was maintained constantly (8 to 9) by the continuous addition of the base solution (30% NH_4_OH). The resulting slurry was aged at 70 °C for 2 h. The solid product was isolated by filtration, washed thoroughly with deionised water (to make the catalyst free from base) and dried at 110 °C for 12 h in oven.

Step 3: the copper supported on MgO/γ-Al_2_O_3_ was further calcined at 750 °C for 4 h to get CuO@Mg/γ-Al_2_O_3_. CuO@Mg/γ-Al_2_O_3_ was then reduced under 3 bar of H_2_ pressure at 350 °C to get the final desired reduced Cu(0)@Mg/γ-Al_2_O_3_ catalyst. Copper nanoparticles anchored on various supports like SiO_2_, TiO_2_, CeO_2_, MoO_3_ and γ-Al_2_O_3_ are also prepared (refer SI for catalyst preparation†) and their performance were checked for FF to FA conversion.

## 3. Results and discussions

### 3.1 Catalyst characterization

#### 3.1.1 X-ray diffraction analysis (XRD)

X-ray diffraction (XRD) of the prepared samples were analysed in the range of 5° ≤ 2*θ* ≤ 80° using Cu Kα radiation (*γ* = 1.5406 Å). The XRD pattern of the samples revealed a highly ordered distinguished two set of peaks as shown in Fig. 2. The diffraction peaks at 43.37°, 50.56° and 74.21° corresponds to (111), (200) and (220) planes, respectively indicating the formation of Cu^0^ from CuO under reduced conditions (JCPDS file no. 04-0836). Peak broadening observed for Cu(0)@Mg/γ-Al_2_O_3_ is consistent with the small particle size ∼5.1 nm. The average crystal sizes calculated using Scherrer formula is 5.0 nm. The XRD results agree well with the HR-TEM analysis indicating that the average particles are constituted of a single crystalline domain. Further, to understand the catalyst stability, high-temperature XRD analysis was performed under atmospheric conditions at various temperatures (Fig. 2B–D).

**Fig. 1:**
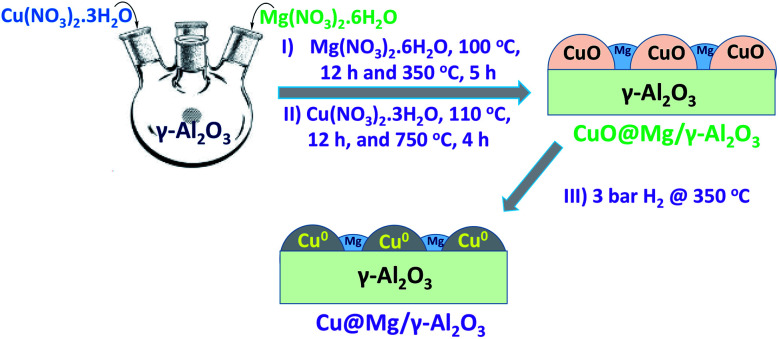
Schematic representation for synthesis of Cu@Mg/γ-Al_2_O_3_.

The catalyst did not show any phase change up to 200 °C and gradual conversion of Cu(0) to CuO is clearly observed after 200 °C. At 300 °C most of the Cu(0) is converted to CuO and the total conversion is observed at 400 °C (Fig. 1D). The XRD patterns of the calcined catalysts did not show any clear peaks corresponding to magnesium species. This could be due to the low concentration of Mg in the sample but it could also be a sign of high dispersion of Mg species, since no characteristics structures are observed.

**Fig. 2:**
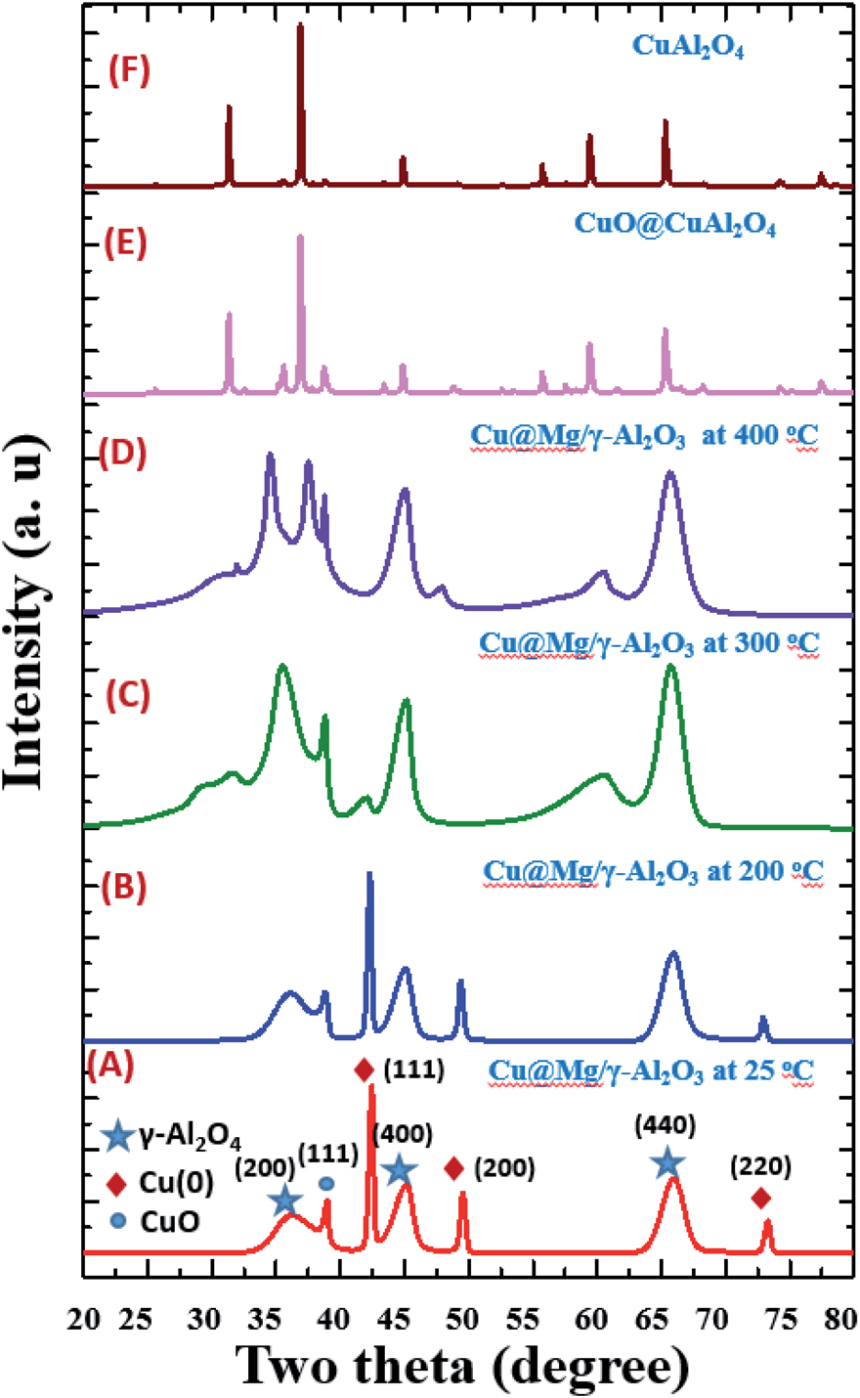
Temperature dependent XRD spectrum of Cu@Mg/γ-Al_2_O_3_ from 25 °C to 400 °C (images A–D), standard CuO@CuAl_2_O_4_ (E) and CuAl_2_O_4_ (F).

#### 3.1.2 HR-TEM and XPS analysis of Cu@Mg/γ-Al_2_O_3_

**Fig. 3:**
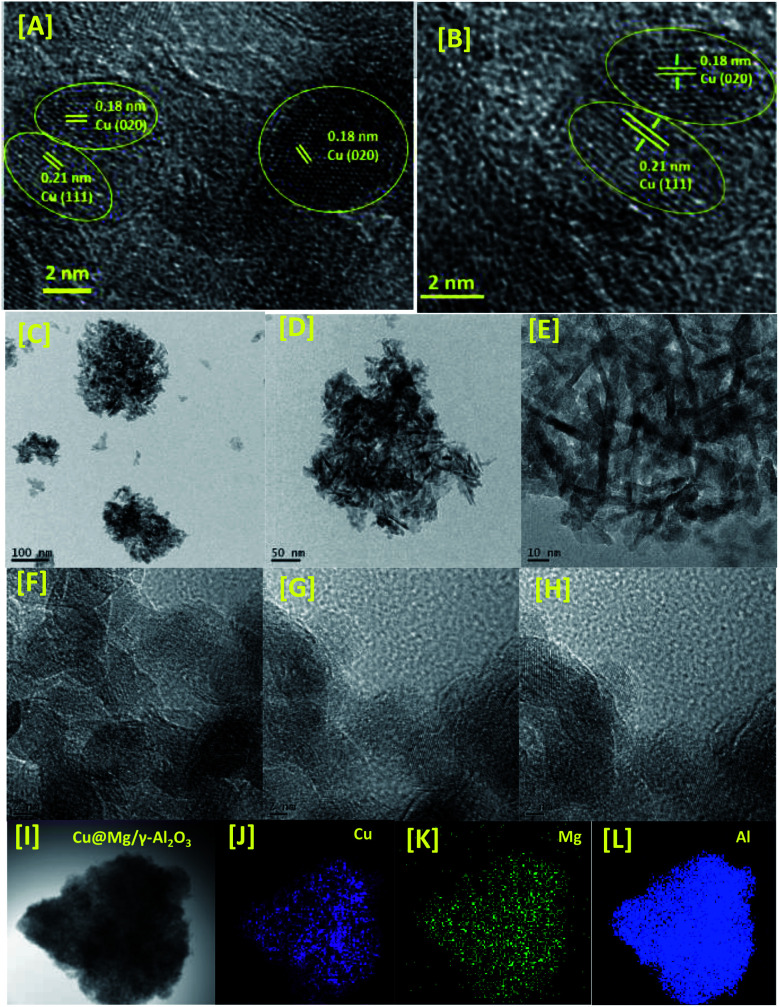
HR-TEM images [A–I] electron diffraction [SAED pattern, B] and elemental mapping [Cu, Mg and Al from J to L] of Cu@Mg/γ-Al_2_O_3_.

To investigate the surface morphology and internal structure of the Cu@Mg/γ-Al_2_O_3_, HR-TEM analysis was conducted (Fig. 3). From HR-TEM it is clearly evident that the copper nanoparticles (Cu^0^) are well dispersed onto the support with the average particle size of copper to be 5 nm. From HR-TEM images it is clear that the formed copper nanoparticles are having two distinguished *d*-spacing of 0.21 and 0.18 nm corresponds to the (111) and (200) lattice planes of the Cu(0). The elemental mapping of the prepared catalyst also confirms the uniform distribution of Cu and Mg on γ-Al_2_O_3_ support.

**Fig. 4:**
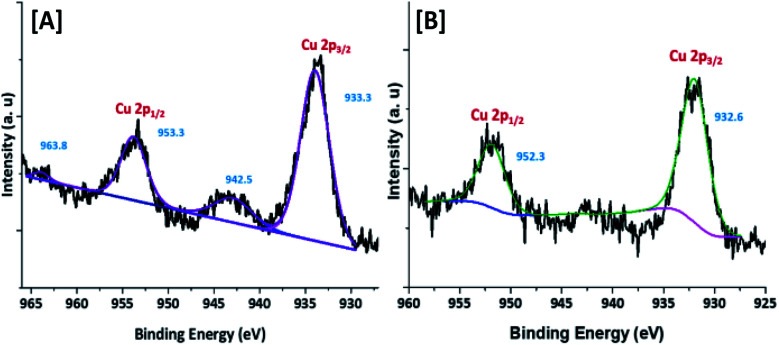
XPS spectrum of [A] Cu@Mg/γ-Al_2_O_3_ and [B] freshly reduced Cu(0)@Mg/γ-Al_2_O_3_.

To confirm the oxidation state and surface composition of the catalyst XPS experiments were recorded. As shown in Fig. 4, the binding energies of Cu@Mg/γ-Al_2_O_3_ was observed at 932.6 and 952.3 eV corresponds to the spin orbit coupling of Cu 2p_3/2_ and Cu 2p_1/2_ which confirmed the metallic nature Cu(0) of copper nanoparticles (Fig. 4B). The surface scanning of Cu@Mg/γ-Al_2_O_3_ by HR-TEM also confirms the uniform distribution of copper throughout the sample (Fig. 3E–H). The peak positions observed at 933.3 and 953.3 eV with the corresponding satellite peaks at 942.5 and 963.8 eV confirmed the Cu 2p core level in the +2 oxidation state (Fig. 4A) of CuO. In contrast, in the case of freshly reduced Cu(0)@Mg/γ-Al_2_O_3_, the binding energies were observed at 932.6 and 952.3 eV corresponding to the zero oxidation state of copper in the reduced catalyst and no satellite peaks at the corresponding positions were observed which strongly suggest the complete reduction of small amount of CuO present in Cu@Mg/γ-Al_2_O_3_ (Fig. 4A) to Cu(0)@Mg/γ-Al_2_O_3_(Fig. 4B). The very low shake satellite peak observed at 942.5 eV and 963.8 eV in Cu@Mg/γ-Al_2_O_3_ catalyst may be due to the small amount of copper oxide (CuO) formed due to copper propensity for aerial oxidation. The formation of Cu(0) nanoparticles by reduction was further confirmed with auger electron microscope (AES) analysis (refer ESI†). Cu@Mg/γ-Al_2_O_3_ (fresh) and reused Cu@Mg/γ-Al_2_O_3_ (recovered after 5^th^ run) were characterized by Auger electron microscopic analysis to confirm the zero oxidation state of the copper, and no oxidation of metallic copper of Cu(0)@Mg/γ-Al_2_O_3_ was found even after successive runs. The kinetic energy for fresh and reused catalyst was found to be 919 eV and 918.7 eV.

### 3.2 Catalytic activity: effect of support

As shown in the Table 1, for the catalytic hydrogenation of FF, copper nanoparticles anchored on various supports like SiO_2_, TiO_2_, CeO_2_, MoO_3_ and γ-Al_2_O_3_ were evaluated under solvent free reaction conditions at different temperatures. Among the screened catalysts, copper nanoparticles supported on γ-Al_2_O_3_ was found to give the best conversion of FF >99% with FA yield of 74% in 5 h, extending the reaction time to 12 h gave further improvement in reaction yield of FA to 83%. γ-Al_2_O_3_ is extensively used as support in various active catalysts because of its unique mechanical and chemical properties^12–15^ and high surface area. γ-Al_2_O_3_ supported catalysts show favourable activity in many reactions such CO_2_ hydrogenation,^16^ for hydrodesulfurization^17^ and reduction of NO_*x*_.^18^ However, the formation of side products could not lead the conversion to maximum yields (Table 1, entries 1 and 2). In case of SiO_2_, the deoxyhydrogenation product, 2-methyl furan (2-MF) is the major product with 40% yield along with only 10% yield of FA. On the other hand, other solid supports such as TiO_2_ resulted in the moderate conversion with less yield (Table 1, entry 4). Next, it was observed that CeO_2_ and MoO_3_ were less effective for the selective hydrogenation of FF to FA (Table 1, entries 5 and 6). Also, we have compared alumina supported precious metal catalysts like Pt, Rh, Ir and Pd with non-precious catalysts, among them Pt has shown good conversion towards deoxyhydrogenated furfural to 2-MF with 88% yield. Rh, Ir and Pd could give decarbonylated product furan as a major product with 87%, 96% and 99% of yields (Table 1, entries 7–10) respectively. Nevertheless, copper metal sites combined with an aluminium oxide site can play a major role in the activity, selectivity and stability of the samples. Thus, aluminium oxide acts mainly as structural and textural promoter, providing a larger surface area for solids containing copper oxide.^18^ The preparation of supported catalyst can be seen in ESI.†

**Table 1:** Hydrogenation of furfural using various supported metal catalysts^a^

Entry	Catalyst	Conv. [%]	Yield [%]				
			FA	2-MF	THFA	Furan	THF
1^b^	Cu/γ-Al_2_O_3_	>99	74	16	3	2	Trace
2^c^^,^^d^	Cu/γ-Al_2_O_3_	>99	83	0	1	4	0
3	Cu/SiO_2_	>60	10	40	Trace	Trace	Trace
4	Cu/TiO_2_	20	15	2	Trace	Trace	Trace
5	Cu/CeO_2_	45	23	Trace	Trace	Trace	Trace
6	Cu/MoO_3_	20	8	Trace	Trace	Trace	Trace
7	Pt/γ-Al_2_O_3_	>90	12	88	Trace	Trace	Trace
8	Rh/γ-Al_2_O_3_	>99	—	—	Trace	87	5
9	Ir/γ-Al_2_O_3_	>99	—	—	—	96	—
10	Pd/γ-Al_2_O_3_	>99	—	—	—	99	—

a Reaction conditions: furfural (2.6 mol%); catalyst (0.1 g, noble metal 2 mol%); H_2_ (2 MPa); 443.15 K; 5 h.

b Analyzed by GC using an toluene as internal standard.

c Catalyst (0.1 g, Cu 0.4 mol%).

d 12 h.

Further, in order to design efficient catalytic system for FF hydrogenation copper nanoparticles supported on bimetallic supported system was examined. Recently, magnesium doped copper and iron based catalysts successfully employed for transfer hydrogenation of furfural using isopropanol as a hydrogen donor.^19^ The increase in activity can be attributed to the change in the electronic and physical structure of the catalyst and in-turn enhances increase in the absorption ability of the reacting molecules on the host material. The high catalytic activity with Mg doped supports can be attributed due to increasing adsorption sites of hydrogen on MgO surface with low coordination sites on the catalyst surface.^20^ Typically, MgO acts as a promoter to improve activity and/or selectivity acting as a basic site to polarise the C=O bond of furfural which in turn facilitate nucleophilic attack by hydrogen dissociatively adsorbed on adjacent Cu active sites. Besides this, the basic sites also help to reduce the concentration of Lewis acid centres on the support γ-Al_2_O_3_, which will decrease coke deriving from the acidic strength of γ-Al_2_O_3_. The same can be witnessed from CO_2_-TPD data wherein basic site concentration is found to enhance in presence of MgO (see ESI for CO_2_-TPD†). The effect of different metals as promoters such as: Co, Zn, Mg, Ga, Mn and Zr on catalytic activity of Cu/γ-Al_2_O_3_ in FA synthesis was also studied (Table 2). Based on the literature reports and in our present investigation to increase the catalytic activity of copper supported catalyst for hydrogenation of FF, magnesium has been doped in various molar ratios and their catalytic activity were investigated (Table 3). MgO increases the strength of the interaction between Cu and Al_2_O_3_ and induces a spillover effect between these phases. Synergistic catalytic effect between the catalytically active metallic copper species and the Lewis basic sites, which held the key to the hydrogenation reaction related to the hydrogen dissociation and the activation of the carbonyl groups attribute to its high catalytic efficiency. Another advantage of using MgO as a promoter for the preparation of Cu@Mg/γ-Al_2_O_3_ catalyst arises from the possibility of forming a CuO–MgO solid solution at any molar ratio due to close ionic radii of Mg^2+^ and Cu^2+^ cations (Mg^2+^ 0.65 Å and Cu^2+^ 0.73 Å) and the particular lattice parameters of this mixed metal oxide structure. The formation of this mixed oxide phase favors increased metal-support interaction, and thus prevents catalyst deactivation *via* sintering. On the other hand, the promotion of copper catalysts by magnesium oxides increases the amount of hydroxylalkyl intermediate group formed on the catalysts surface during the process compared to the reaction carried out with the unprompted catalysts. ^1^H NMR analysis of the reaction intermediate showed the formation of hydroxyalkyl intermediate in 9% yield, which transformed to FA with Cu–Mg/γ-Al_2_O_3_.

**Table 2:** Promoter effect in conversion of furfural to furfuryl alcohol^a^

Sr. no.	Bimetallic catalyst	FF conv. [%]	FA yield^b^ [%]				
			FA	2-MF	Furan	THF	THFA
1	Cu–Co/γAl_2_O_3_	65	45	4	0	0	5
2	Cu–Zn/γAl_2_O_3_	90	70	16	4	1	0
3	Cu–Mg/γAl_2_O_3_	>99	94	2	4	0	0
4	Cu–Ga/γAl_2_O_3_	75	15	3	0		17
5	Cu–Mn/γAl_2_O_3_	80	32	8	5	3	0
6	Cu–Zr/γAl_2_O_3_	80	40	0	0	0	0

a Reaction conditions: furfural (2.6 mol%); catalyst (0.1 g, noble metal 2 mol%); H_2_ (2 MPa); 443.15 K; 5 h. In all the bimetallic catalysts, Cu/X metal ratios were maintained as 5 (10 wt% copper and 2 wt% X metal {X = Co, Zn, Mg, Ga, Mn and Zr}) on the support γ-Al_2_O_3_.

b Analyzed by GC using toluene as an internal standard.

**Table 3:** Cu–Mg ratio effect in conversion of furfural to furfuryl alcohol^a^

Sr. no.	Cu/Mg	FF conv. [%]	FA yield^b^ [%]				
			FA	2-MF	Furan	THF	THFA
1	4	>99	90	3	2	1	1
2	5	>99	94	2	4	0	0
3	10	>99	80	15	0	0	0
4	15	>99	65	22	2	0	1

a Reaction conditions: furfural (2.6 mol%); catalyst (0.1 g, Cu/Mg mol%); Cu/Mg = 4 (Cu: 0.2 mol% and Mg: 0.05 mol%); H_2_ (2 MPa); 443.15 K; 5 h.

b Analyzed by GC using toluene as an internal standard.

Among all, Cu–Mg supported catalysts showed the most superior activity performance in terms of FF conversion, FA formation rate, and yield. Therefore, the Cu–Mg was employed hereafter to compare with other support materials (Table 4). In the case of γ-Al_2_O_3_, FF conversion attributed 90% conversion with 85% of FA product yield, increase in reaction time to 5 h further increases the conversion to 99% with 94% of product selectivity (Table 4, entries 1 and 2). On the other hand, other solid supports such as SiO_2_, TiO_2_, CeO_2_ and MoO_3_ resulted in moderate yields with 2-MF and furan as a major side products (entries 8 to 11). The support γ-Al_2_O_3_ with high surface area than other supports (Table 2, entries 8 to 11) shows highly dispersed copper species achieved the highest catalytic activity compared to the sample containing copper on SiO_2_, TiO_2_, CeO_2_ and MoO_3_. The presence of γ-Al_2_O_3_ interacting with copper oxide provides a lower sintering under optimized reaction conditions.^21^ It is also clearly noticeable that the magnesium doped catalysts are comparatively higher in activity than magnesium free catalysts (Table 4, entries 1–5 *vs.* 6). These results clearly show that the combination of Cu(0) nanoparticles and Mg on γ-Al_2_O_3_ support is uniquely effective for the synthesis of FA from FF under solvent-free conditions. Furthermore, the addition of a second metal MgO as promoter has been found to improve activity and/or selectivity by acting as a basic site to polarise the <svg xmlns="http://www.w3.org/2000/svg" version="1.0" width="10.400000pt" height="16.000000pt" viewBox="0 0 10.400000 16.000000" preserveAspectRatio="xMidYMid meet"><metadata> Created by potrace 1.16, written by Peter Selinger 2001-2019 </metadata><g transform="translate(1.000000,15.000000) scale(0.011667,-0.011667)" fill="currentColor" stroke="none"><path d="M80 1160 l0 -40 40 0 40 0 0 -40 0 -40 40 0 40 0 0 -40 0 -40 40 0 40 0 0 -40 0 -40 40 0 40 0 0 -40 0 -40 40 0 40 0 0 -40 0 -40 40 0 40 0 0 -40 0 -40 40 0 40 0 0 80 0 80 -40 0 -40 0 0 40 0 40 -40 0 -40 0 0 40 0 40 -40 0 -40 0 0 40 0 40 -40 0 -40 0 0 40 0 40 -40 0 -40 0 0 40 0 40 -80 0 -80 0 0 -40z M560 520 l0 -40 -40 0 -40 0 0 -40 0 -40 -40 0 -40 0 0 -40 0 -40 -40 0 -40 0 0 -40 0 -40 -40 0 -40 0 0 -40 0 -40 -40 0 -40 0 0 -40 0 -40 -40 0 -40 0 0 -40 0 -40 80 0 80 0 0 40 0 40 40 0 40 0 0 40 0 40 40 0 40 0 0 40 0 40 40 0 40 0 0 40 0 40 40 0 40 0 0 40 0 40 40 0 40 0 0 80 0 80 -40 0 -40 0 0 -40z"/></g></svg> C=O bond facilitating nucleophilic attack by hydrogen dissociatively adsorbed on neighbouring Cu active sites. Moreover, with a capacity for oxygen storage, MgO can release oxygen to oxidize the carbon formed on the catalyst surface. Particularly, the presence of the basic centre will strengthen the chemisorption of carbonyl group. Next, to understand the importance of hydrogen pressure, the reaction was performed at 1 MPa, which took almost 10 h to complete the conversion of FF to FA with 93% selectivity of desired product (Table 4, entry 5). As evident from Tables 1 and 2 the optimum condition for hydrogenation of furfural is at 443.15 K for 5 h at 2 MPa to get maximum yield of FA.

**Table 4:** Effect of supports on the hydrogenation of furfural using Cu–Mg catalysts^a^

S. no.	Catalyst	H_2_ [MPa]	Time [h]	FF conv. [%]	Yield [%]				
					FA	2-MF	Furan	THF	THFA
1	Cu–Mg/γ-Al_2_O_3_	2	3	90	85	0	3	Trace	0
2^b^	Cu–Mg/γ-Al_2_O_3_	2	5	>99	94	2	4	0	0
3^c^	Cu–Mg/γ-Al_2_O_3_	2	5	>99	94	2	4	0	0
4	Cu–Mg/γ-Al_2_O_3_	2	4	>99	90	4	4	1	0
5	Cu–Mg/γ-Al_2_O_3_	1	10	>99	93	Trace	5	2	0
6	Cu/γ-Al_2_O_3_	2	10	>99	74	16	3	Trace	2
7	MgO/γ-Al_2_O_3_	2	10	Trace	0	Trace	0	Trace	0
8	Cu–Mg/SiO_2_	2	5	>99	44	0	26	9	17
9	Cu–Mg/TiO_2_	2	5	>99	39	49	8	4	Trace
10	Cu–Mg/CeO_2_	2	5	>99	2	53	0	0	0
11	Cu–Mg/MoO_3_	2	5	>99	32	Trace	0	0	0

a Reaction conditions: FF (2.6 mol%); catalyst (2 g, Cu–Mg: Cu@Mg, Cu-0.2 mol%, Mg 0.05 mol%); H_2_ (2 MPa); 443.15 K.

b Cu/Mg = 5.

c Reuse 3.

### 3.3 Catalyst stability and reuse experiments

**Fig. 5:**
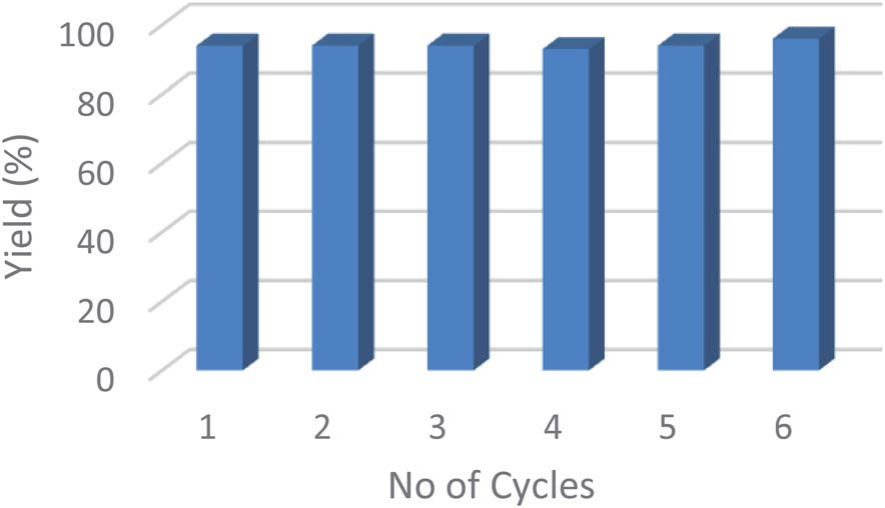
Recyclability of the catalyst for reduction of furfural up to six consecutive runs.

From an industrial perspective, one of the main advantages of using heterogeneous catalysts such as Cu@Mg/γ-Al_2_O_3_, is that they can be recovered and reused efficiently up to six consecutive runs (Fig. 5). The spent catalyst was recovered from the reaction mixture by simple centrifugation after the completion of the reaction and washed with EtOAc (3 × 10 mL) to remove all the organic substrates from the catalyst surface. No quantifiable amount of leached Cu was detected in the filtrate as determined by ICP (AES) studies of both fresh and spent catalyst. Furthermore, the HR-TEM images of the used catalyst did not show any significant change in the shape and size of the support as well as the particle size of the active species, Cu(0). This suggests that the morphology of the catalyst remains the same even after multiple reaction cycles Table 5.

**Table 5:** Recyclability of Cu@Mg/γ-Al_2_O_3_ for hydrogenation of FF up to five runs^a^

Reaction run	Run 1	Run 2	Run 3	Run 4	Run 5	Run 6
FA yield (%)	94	94	94	93	94	96

a Reaction conditions: FF (2.6 mol%); Cu@Mg/γ-Al_2_O_3_ (0.1 g); 443.15 K; H_2_ (2 MPa); 5 h; yields analyzed by GC using toluene as an internal standard.

### 3.4 Representative reaction procedure

The hydrogenation of furfural was carried out in a 500 mL stainless steel autoclave PARR reactor. The vessel was charged with 2.6 mol of fufural, and 0.1 g of catalyst. The reactor was sealed, purged three times with H_2_ at 2 MPa, then pressurized to 2 MPa, heated to 443.15 K and stirred at 300 rpm for 5 h with continuous H_2_ flow (80 SLPH). Following the reaction, the autoclave was cooled to room temperature and the hydrogen gas was carefully released. The resulting reaction mixture was centrifuged and catalyst was separated from the reaction. The reaction mixture is then diluted with Toluene and analyzed by GC. Cu@Mg/γ-Al_2_O_3_ catalyst provides FA product in good to excellent yields over short reaction times compared with reported catalyst in the absence of solvents and strong bases (see ESI Table 1†). Furthermore, the catalyst was applicable to preparative scale reaction; 500 g of FF afforded 502 g of FA in 96% isolated yield (ESI†).

## 4. Conclusions

The present study demonstrates high activity, selectivity and stability of chromium-free Cu-based catalyst supported on MgO-doped γ-Al_2_O_3_ in solvent free hydrogenation of furfural to furfuryl alcohol. The prepared catalyst is found to be selective in the hydrogenation of furfural to furfuryl alcohol with selectivity of 95% at 100% conversion of furfural. The remarkably high performance of this catalyst is attributed to the synergistic effect of copper and magnesium resulting in active Cu–Mg species on the surface of alumina.

## Conflicts of interest

There are no conflicts to declare.

## Supplementary Material

RA-010-D0RA08754H-s001
