# Supplementary material for: Chromium-free Cu@Mg/γ-Al2O3 – an active catalyst for selective hydrogenation of furfural to furfuryl alcohol
Source: RSC Adv. 2020 Nov 10;10(67):41120–6. doi: 10.1039/d0ra08754h (PMC9057799; doi:10.1039/d0ra08754h)
Supplement: RA-010-D0RA08754H-s001 [file RA-010-D0RA08754H-s001.pdf]

Electronic Supplementary Material (ESI) for RSC Advances.
This journal is © The Royal Society of Chemistry 2020

Chromium-free Cu@Mg/γ-Al2O3 an Active Catalyst for Selective Hydrogenation of Furfural to Furfuryl Alcohol

Arundhathi Racha[a], Panyala Linga Reddy[b], Chanchal Samanta[a] and Bharat L Newalkar[a*]

[a] Corporate Research & Development Centre, Bharat Petroleum Corporation Limited, Greater Noida, Uttar Pradesh-201306, India.
[b] Department of Chemistry, Indian Institute of Technology Bombay, Powai, Mumbai-400076, India.

Contents

**1**	General remarks	2
**2**	Experimental (Catalyst Preparation)	3
**2a**	Mono metallic supported catalyst preparation	3
**2b**	Various bimetallic supported catalyst preparation	4
**3**	Catalyst Characterization	5
**3a**	BET surface area, Pore Volume, Pore Size and Particle Size	5
**3b**	Temperature Programmed Reduction (TPR)	7
**3c**	Temperature Programmed Desorption (TPD)	9
**3d**	High Resolution Transmission Electron Microscopy (HR TEM) Analysis	11
**3e**	XPS and AES analysis of Cu@Mg/γ-Al_2O_3	14
**3f**	XRD analysis of synthesized catalysts	16
**4**	General Procedure for the Hydrogenation Reaction	20
**5**	Reaction Kinetics for the Hydrogenation Reaction	20
**6**	Inductively Coupled Plasma-Atomic Emission Analysis (ICP-AES)	21
**7**	^{1}H and ^{13}C NMR spectrum of Furfuryl alcohol:	22
**8**	References	23

Experimental

1) General Information

All reagents were purchased from commercial suppliers and used without further purification. All hydrogenation experiments were carried out under hydrogen (purity: 99.95%). Column chromatography was carried out with Merck silica gel 60-120 mesh and the products were visualized by GC detection. ^{1}H NMR and ^{13}C NMR (Bruker (Germany) Avance III) spectra were recorded in $CDCl_3$. Chemical shifts (δ) are reported in ppm using TMS as an internal standard, and spin -spin coupling constants (J) are given in Hz. ^{1}H NMR and ^{13}C NMR of the compounds were proved either by comparison to the known compounds or the synthesized compounds according to the literature. BET surface area was obtained with N_2 as adsorbate at liquid nitrogen temperature in Micromeritics ASAP-2020 plus. Hydrogen Temperature Programmed Reduction (H_2-TPR) and Carbon dioxide Temperature Programmed Desorption (CO_2-TPD) analysis of the prepared copper catalysts were performed on Autochem II 2920. The particle size and external morphology of the samples were observed on a JEOL JEM-2100 high resolution transmission electron microscope (HR-TEM). XPS spectra were recorded on a Kratos AXIS supra with a dual anode (Mg and Al) apparatus using the Mg Kα anode. The pressure in the spectrometer was about 10^{-9} Torr. For energy calibration, we have used the carbon 1s photoelectron line. The carbon 1s binding energy was taken to be 284.8 eV. The location and the full width at half maximum (FWHM) for a species were first determined using the spectrum of a pure sample. The location and FWHM of the products, which were not obtained as pure species, were adjusted until the best fit was obtained. Symmetric Gaussian shapes were used in all cases. Auger electron spectroscopic (AES) analysis is conducted, at a base pressure of 10^{-10} Torr, within the K.E. range of 110-700 eV (beam voltage of 3 kV, eV/step 1 eV, time/step 50 ms). Powdered X-ray

powder diffraction (PXRD) data were collected on AERIS PANanalytical diffractometer using Cu Kα radiation.

2.0 Catalyst Preparation:

2a. Mono metallic supported catalyst preparation:

Cu/γ-Al_2O_3: In a 250 mL round bottomed flask 100 mL of deionized water was taken and 22.11 gram of $Cu(NO_3)_2.3H_2O$ (0.0915 moles) was added under stirring conditions at room temperature for complete dissolution of copper salt. To this solution, 20 gram of calcined γ-Al_2O_3 support added and stirred at room temperature for 2 hours. The pH of the reaction mixture was maintained constantly (8 to 9) by the continuous addition of the base solution (30% NH_4OH). The resulting slurry was aged at 70 ^oC for two hours. The solid product was isolated by filtration, washed thoroughly with deionised water (to make the catalyst free from base) and dried at 110 ^oC for 12 h in oven.

The copper supported on γ-Al_2O_3 was further calcined at 773 K for 3 h to get CuO /γ- Al_2O_3. CuO@γ-Al_2O_3 was then reduced under 3 bar of H_2 pressure at 350 °C to get the final desired reduced Cu(0)/γ-Al_2O_3 catalyst. Copper(0) anchored on various supports like SiO_2, TiO_2, CeO_2, and MoO_3 were also prepared in similar procedure as described above and their performance were checked in hydrogenation of furfural to furfuryl alcohol.

Pt/ γ-Al_2O_3: 4 mL of an aqueous solution of H_2PtCl_6 (50 mM) was added to 50 mL of distilled water at 25 ^oC. Then, γ-Al_2O_3 (1 g) was added to the solution and the mixture was stirred magnetically while maintaining the same temperature for 4 h. Water was removed by rotary evaporation under reduced pressure to give the solid product. The obtained powder was dried at 110 ^oC for 5 h. Then, calcined at 500 ^oC for 3 h under a static air atmosphere to obtain Pt/γ-Al_2O_3 as a dark gray powder. ICP-AES analysis determined that the γ-Al_2O_3 had been impregnated with Pt at concentrations of 4 wt%. γ-Al_2O_3-supported monometallic catalysts were prepared by the impregnation method using various noble metal salts. The noble metal

contents were adjusted to 0.02 mol of noble metal/g catalyst. In the case of Pt/γ-Al_2O_3, Pt content was 4.2 wt%.

2b. Various bimetallic supported catalyst preparation:

Step 1: 200 mL of deionised water was taken in a 1 L four neck round bottom flask and equipped with an overhead mechanical stirrer. $Mg(NO_3)_2.6H_2O$ (6.63 g, 0.0447 moles) was dissolved in the solution and 45 grams of γ-Al_2O_3 (45 g, 0.4413 moles) was added to $Mg(NO_3)_2.6H_2O$ dissolved solution. The resulting slurry was kept for stirring for 2 hours and aged at 70 °C for 4h. The solid product was isolated by filtration and dried at 110 °C for 12 h in an air oven. Mg doped alumina catalyst was then calcined at 350 °C in the presence of air for 5 hours to obtain MgO/γ-Al_2O_3 and then cooled to room temperature.

Step 2: In a 250 mL round bottomed flask 100 mL of deionized water was taken and 22.11 gram of $Cu(NO_3)_2.3H_2O$ (0.0915 moles) was added under stirring conditions at room temperature for complete dissolution of copper salt. To this solution, 20 gram of calcined MgO/γ-Al_2O_3 catalyst added and stirred at room temperature for 2 hours. The pH of the reaction mixture was maintained constantly (8 to 9) by the continuous addition of the base solution (30% NH_4OH). The resulting slurry was aged at 70 °C for two hours. The solid product was isolated by filtration, washed thoroughly with deionised water (to make the catalyst free from base) and dried at 110 °C for 12 h in oven

Step 3: The copper supported on MgO/γ-Al_2O_3 was further calcined at 750 °C for 4 h to get CuO@Mg/γ-Al_2O_3. CuO@Mg/γ-Al_2O_3 was then reduced under 3 bar of H_2 pressure at 350 °C to get the final desired reduced Cu (0)@Mg/γ-Al_2O_3.

Copper and other bimetallic catalyst anchored on γ-Al_2O_3 is prepared in similar procedure and their performance were checked for FF to FA conversion.

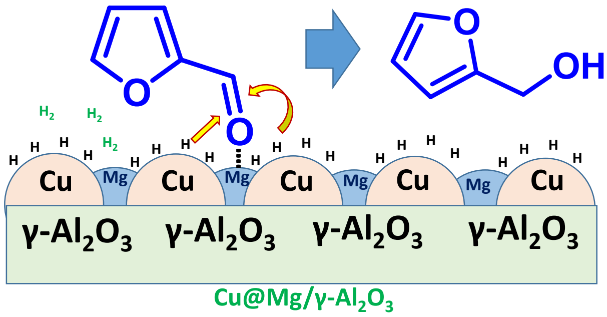

Figure 1. Schematic representation of Cu@Mg/γ-Al_2O_3

Table 1. ICP-AES analysis of catalyst Cu@Mg/γ-Al_2O_3.

S. No	Catalyst	ICP-AES (Cu & Mg mol%)
1[a]	Cu@Mg/γ-Al_2O_3	Cu: 0.2001 & Mg: 0.0514
2[b]	Reaction filtrate	Cu: 0.0001 & Mg: trace
3[c]	Cu@Mg/γ-Al_2O_3	Cu: 0.2000 & Mg: 0.0511

[a]Fresh catalyst, [b] Reaction filtrate (catalyst removed by filtration), [c] Reaction with fresh catalyst .

3. Catalyst Characterization.

3a. BET surface area, Pore Volume, Pore Size and Particle Size:

N_2 adsorption–desorption isotherms and pore size distribution of the prepared catalysts are shown in figure 2. The adsorption isotherms of γ-Al_2O_3 and Cu@Mg/γ-Al_2O_3 catalysts are of type IV with a H_2 hysteresis loop at relative pressure ranging from 0.6 to 1.0, which is the characteristic of ink-bottle pores. However, the hysteresis loops of Cu@Mg/γ-Al_2O_3 are smaller than that of γ-Al_2O_3 carrier, owning to the insertion of active component in the pore channels (Figure 2D). As shown in the pore size distribution curves, the pore size distributed mainly at 5.0 nm, which is favorable for the diffusion of reactant furfural. The specific surface area and pore volume were decreased after the active component was loaded over the carrier. This is because that the active component can occupy a part of surface area and the pore structure would collapse to a certain extent. The active components could insert into the pore channel of γ-Al_2O_3 carrier, which would transform into metal oxide during the calcination and block a part of pores, resulting in the decrease of specific surface area. In addition, the pores

collapsed partly during the calcination process, which could also decrease the specific surface area. All catalysts have similar surface area and pore volume as they have same active component loading. They all have an average pore size of 6.0 nm, which is favor to the diffusion of reactant during the hydrogenation process. In addition, large surface area can provide much more active sites for hydrogenation, as the reaction mainly occurred on the surface of the catalysts. The overall increase in surface area in Cu@Mg/γ-Al_2O_3 than Cu/γ-Al_2O_3 can be attributed to an increase in specific surface area of the active metal with an addition of Mg as promoter.

Table 2. Textural properties of Cu-Mg/ γAl_2O_3.[a]

Sr. No	Catalyst	BET Surface area (m^2/g)	Pore size (nm)	Total pore volume(cc/g)
1	γ-Al_2O_3	230	7.8	0.73
2	MgO/γ-Al_2O_3	200	5.8	0.70
3	Cu/γ-Al_2O_3	180	5.5	0.72
4	Cu@Mg/γ-Al_2O_3	200	5.0	0.69

[a]MgO/γ-Al_2O_3, Mg: 0.2 mol%; Cu/γ-Al_2O_3, Cu: 0.25 Mol% anad Cu@Mg/γ-Al_2O_3: Cu: 0.25 mol% and Mg: 0.05 mol%

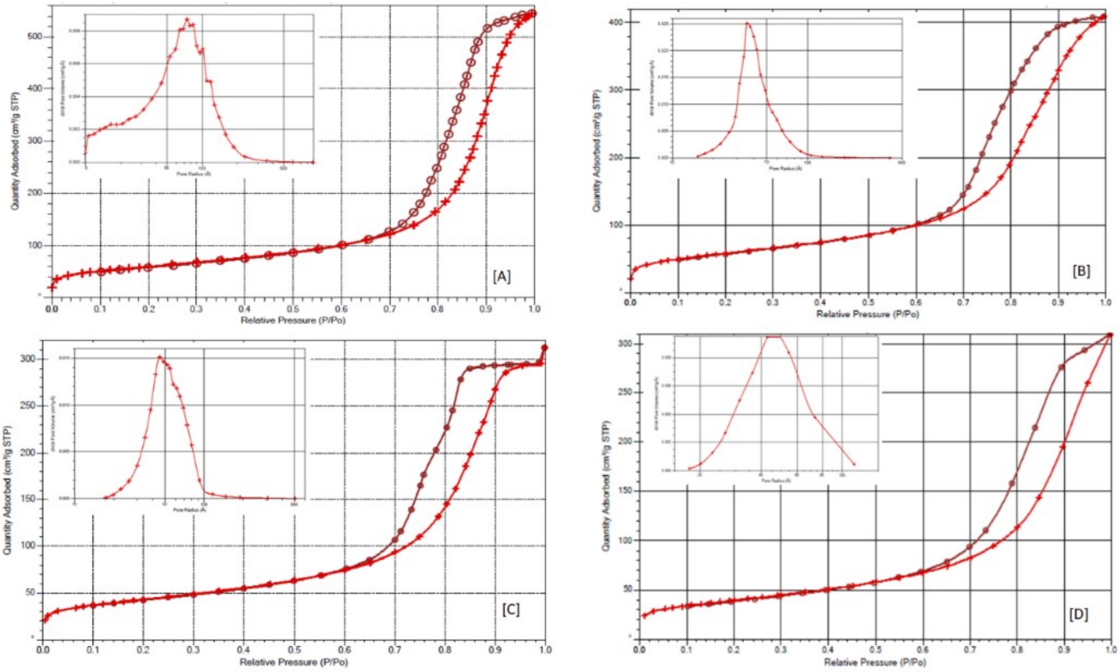

Figure 2. [A] γ-Al_2O_3 [B] MgO/γ-Al_2O_3 [C] Cu/γ-Al_2O_3 [D] Cu@Mg/γ-Al_2O_3.

Table 3. BET analysis of monometallic catalysts on various supports.

Entry	Catalyst	BET (m^2/g)	ESA (m^2/g)	MiPA (m^2/g)	TPV (cc/g)
**1**	Cu/γ-Al_2O_3	180	175	5	0.7201
**2**	Cu/SiO_2	134	120	14	0.495
**3**	Cu/TiO_2	29	19	10	0.076
**4**	Cu/CeO_2	55	53	2	0.043
**5**	Cu/MoO_3	11	8	3	0.076
**6**	Pt/γ-Al_2O_3	187	182	5	0.697
**7**	Rh/γ-Al_2O_3	192	183	9	0.717
**8**	Ir/γ-Al_2O_3	193	185	8	0.698
**9**	Pd/γ-Al_2O_3	192	187	5	0.709

Table 4. BET analysis of bimetallic catalysts.

Entry	Support	BET (m^2/g)	ESA (m^2/g)	MiPA (m^2/g)	MiPV (cc/g)	TPV (cc/g)
1	γ-Al_2O_3	230	200	30	0.0060	0.7302
2	Cu-Co/γ-Al_2O_3	191	166	25	0.0105	0.7317
3	Cu-Zn/γ-Al_2O_3	165	157	8	0.0039	0.7219
4	Cu-Mg/γ-Al_2O_3	200	189	11	0.0059	0.6905
5	Cu-Ga/γ-Al_2O_3	172	159	13	0.0069	0.7095
6	Cu-Mn/γ-Al_2O_3	172	168	4	0.0020	0.7388
7	Cu-Zr/γ-Al_2O_3	182	166	16	0.0087	0.7598

3b. Temperature Programmed Reduction (TPR)

The hydrogen temperature-programmed reduction (H_2-TPR) analysis of the prepared copper catalysts was performed on Micromeritics Autochem II 2920. In a typical experiment, around 0.15 g of catalyst was placed in a quartz sample cell, the sample was then flushed with pure argon at a flow rate of 50 mL min^{-1} and 573 K for 0.5 h and then cooled down to room temperature. Subsequently, a 10% H_2/Ar (30 mL min^{-1}) was flown through the sample while

the temperature was increased to 600 ^{o}C at 10 ^{o}C min^{-1} ramp rate and held at final temperature for 0.5 h. H_2-TPR measurement of Cu/γ-Al_2O_3 and Cu@Mg/γ-Al_2O_3 catalysts were conducted to study the redox properties of calcined samples and the interaction between the copper species and the support. Obviously, two kinds of copper species exist in the CuO-MgO/γ-Al_2O_3 sample figure 3. The broad low-temperature reduction peak at ca. 225 °C is assigned to the reduction of isolated highly-dispersed CuO species, while the sharp high-temperature one at ca. 279 °C is associated with the reduction of Cu^{2+} species having a strong interaction with the matrix.[1-2] Whilst in Cu/γ-Al_2O_3 sample figure 4 two reduction temperature peaks disappears to one temperature 268 ^{o}C and becomes broader. This could be due to the presence of only Cu-containing species and the absence of interaction with MgO matrix, which thus improves the reducibility of copper species. The reduction temperature peak appears well before 350 °C may be due to the reduction of highly-dispersed copper oxide particles. The lower metal loading on the support may have led to the higher dispersion. This is confirmed by TEM images. Chang and coworkers [3] reported that supported samples with low metal loading are easier to reduce.

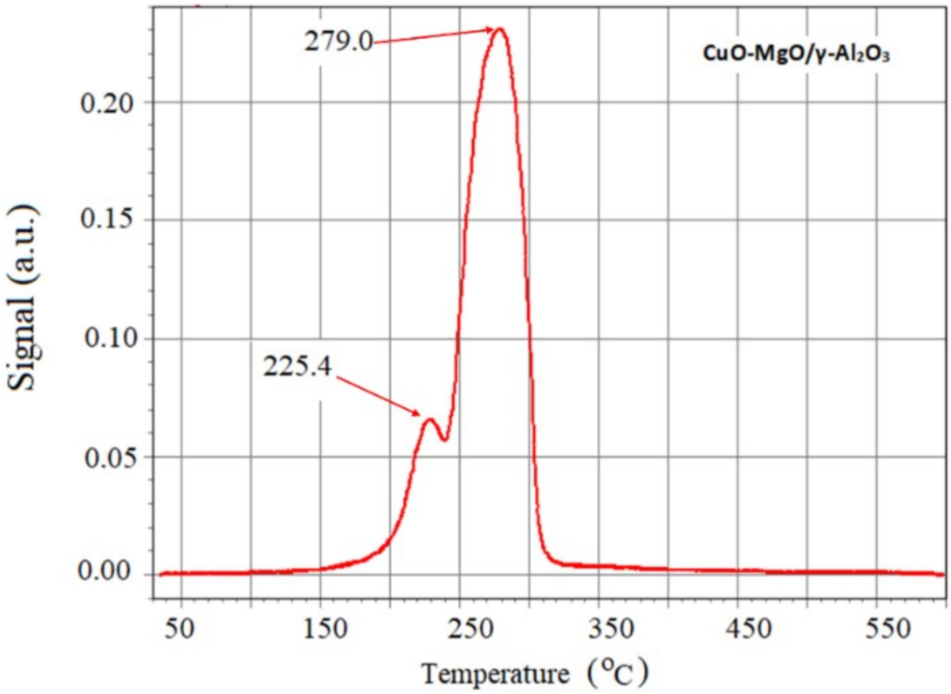

Figure 3. TPR spectrum of calcined CuO@Mg/γ-Al_2O_3.

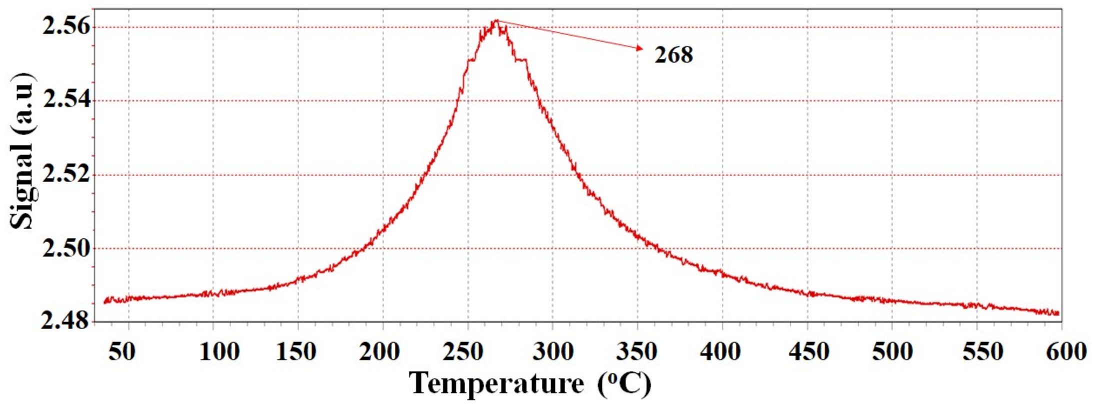

Figure 4. H_2-TPR of Cu/γ-Al_2O_3

Table 5. H_2-Temperature programmed reduction (H_2-TPR) of catalysts.

Sr.No	Catalyst	Temperature (°C)	H_2 consumption (μmol/g)
1	CuO-MgO/γ-Al_2O_3	225.4	294
		279.0	173.8
2	Cu/γ-Al_2O_3	268	138.7

3c. Temperature Programmed Desorption (TPD)

The basic property of the catalysts were measured by temperature programmed desorption of CO_2 equipped with TCD detector. 100 mg of the catalysts were placed in a quartz reactor. The sample was pretreated at 400 °C in He of 60 mL/min for 180 min, and cooled down to room temperature. Then the sample was exposed in CO_2 (30 mL/min) using a six-way valve for 120 min and then flushed with He (40 mL/min) flow to remove all the physically adsorbed CO_2. Once the physically adsorbed CO_2 was purged off, the CO_2-TPD experiments were started. The data was collected at heating rate at 10 °C/min from room temperature to 750 °C in a flow of He (50 mL/min).

Basicity of reduced supported copper catalysts was determined by CO_2-TPD and profiles are shown in figure 5. The CO_2-TPD profile of Cu/Al_2O_3, shows a single desorption at T_{max} of 108.4 °C was ascribed to the weak basic sites. Whereas, Cu@Mg/γ-Al_2O_3 catalyst had two CO_2 desorption peaks at a T_{max} of 111 °C and 387 °C, corresponding to the presence of weak and

moderate basic sites, respectively. The basicity is as follows in the decreasing order: Cu/γ-Al_2O_3 85.8 µmolg^{-1} < Mg-Al-HT 94 < Cu-Mg-Al-HT 96.7 < γ-Al_2O_3 128.3 < MgO/γ-Al_2O_3 129.5 < Cu@Mg/γ-$Al_2O_3$142.5 µmolg^{-1}.

Table 6. CO_2-Temperature programmed desorption (CO_2-TPD) of catalysts

Sr. No	Catalyst	Temperature (°C)	CO_2 Consumption (µmol/g)
1	γ-Al_2O_3	103	128.3
2	Cu/γ-Al_2O_3	108.4	85.8
3	MgO/γ-Al_2O_3	116.5<br>484	82.5<br>47
4	Cu@Mg/γ-Al_2O_3	111<br>387	92.0<br>50.5
5	Mg-Al-Hydrotalcite	115.6<br>387.0<br>677.1	70.3<br>21.8<br>1.9
6	Cu-Mg-Al-Hydrotalcite	113<br>239.5	93.8<br>2.9

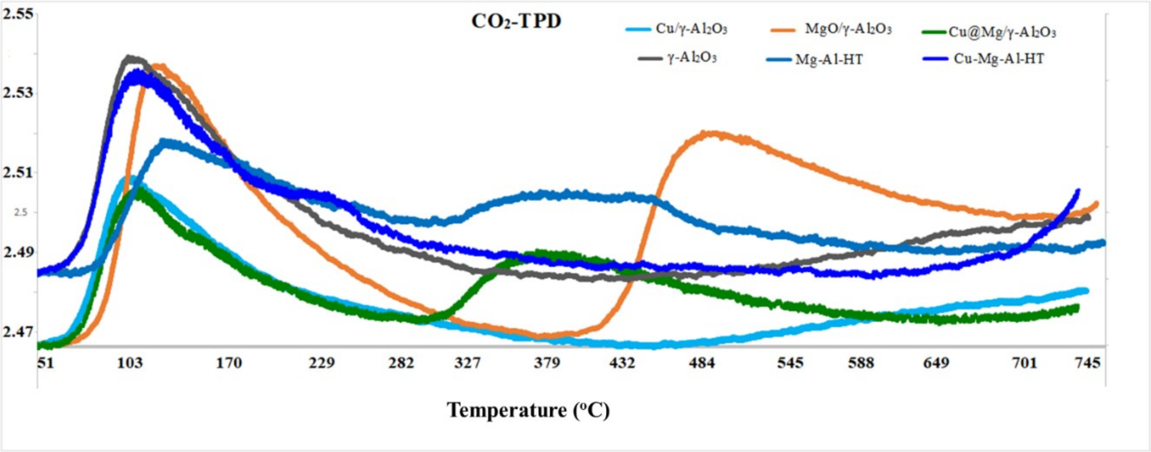

Figure 5. CO_2-TPD of- γ-Al_2O_3;-Cu/γ-Al_2O_3; -Cu@Mg/γ-Al_2O_3 ; -MgO/γ-Al_2O_3; -Mg-Al-HT and -Cu-Mg-Al-HT.

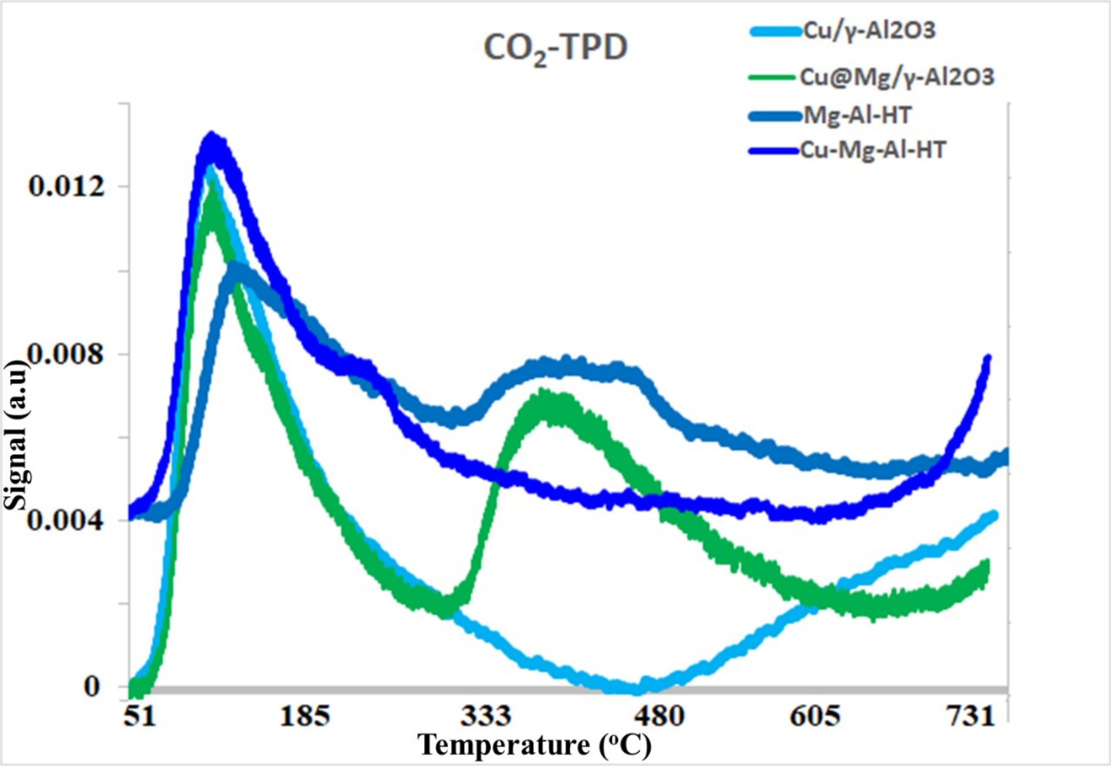

Figure 6. CO_2-TPD of -Cu/γ-Al_2O_3; -Cu@Mg/γ-Al_2O_3; -Mg-Al-HT and -Cu-Mg-Al-HT.

3d. High Resolution Transmission Electron Microscopy (HR TEM) Analysis

HR-TEM analysis of the Cu@Mg/γ-Al_2O_3 catalyst was performed using FEG-TEM-200 kV (JEM 2100F) and found that the copper nanoparticles on the γ-Al_2O_3 are well dispersed with uniform distribution as shown in figure 7. However, the particles are very close to each other can be observed distinguished boundaries of every particle. Further, to check the particle size distribution, we have analysed hundred nanoparticles diameter and plotted the diagram as shown in figure 8. From the images it is also clearly evident that not only dispersion, along with that the size of majority of the nanoparticles are in the range between 3-8 nm with average particle size of 6 nm.

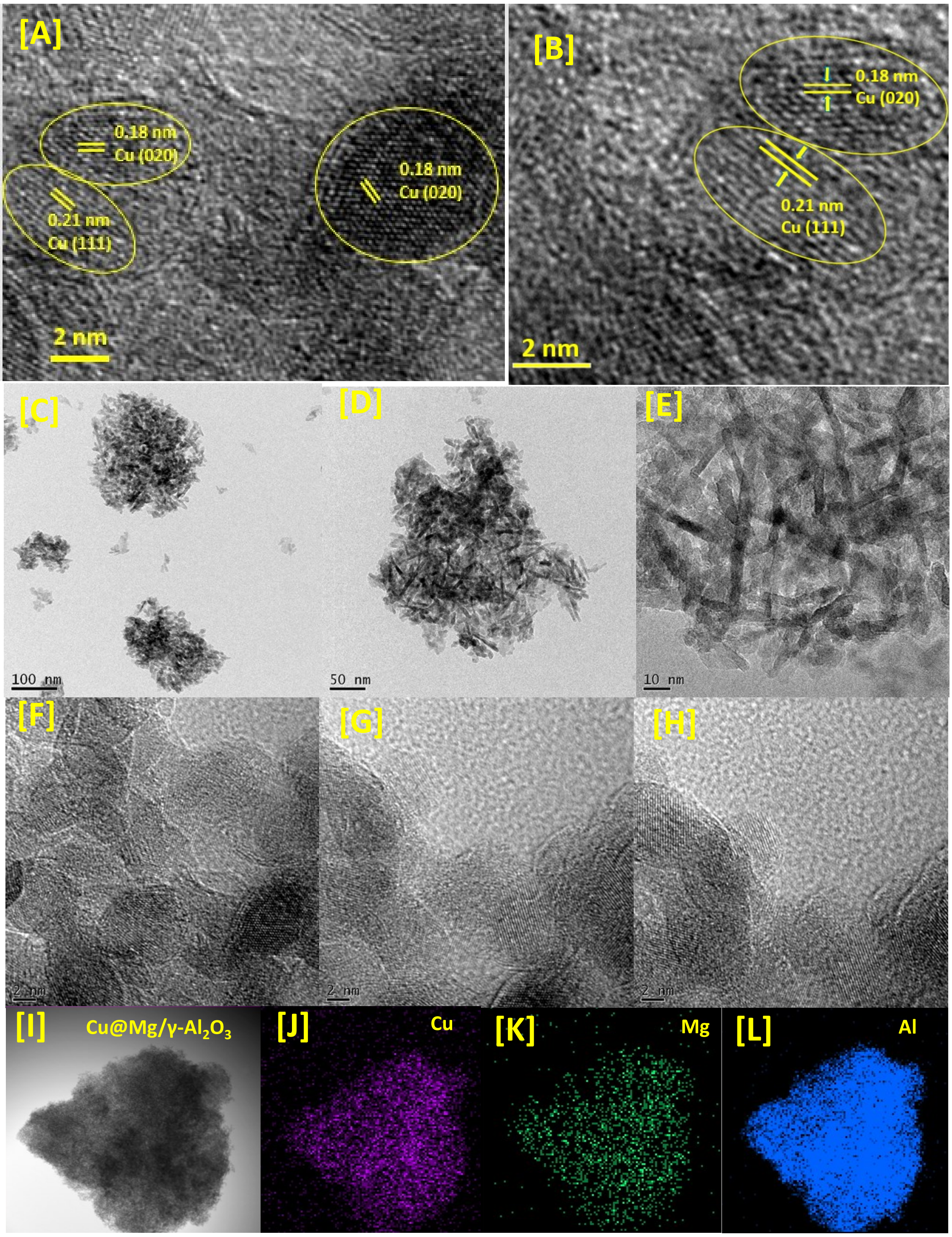

Figure 7. HR-TEM images [A] electron diffraction [SAED pattern, B] and elemental mapping [Cu, Mg and Al from J to L] of Cu@Mg/γ-Al2O3

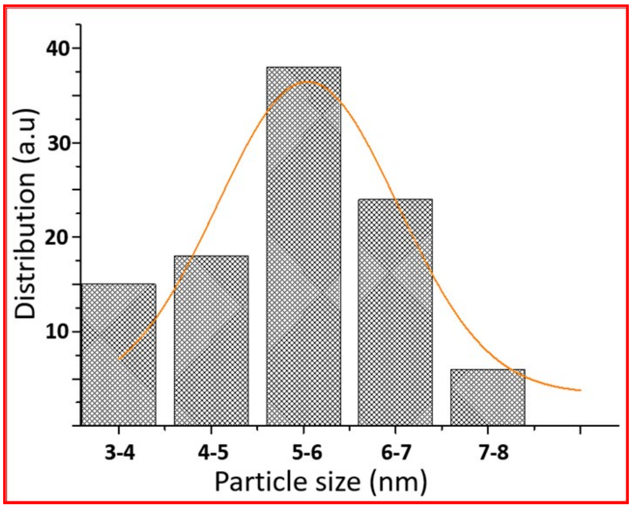

Figure 8. TEM histogram of Cu@Mg/γ-Al_2O_3

3e. XPS analysis of Cu@Mg/γ-Al_2O_3

From the XPS analysis, the peak positions observed at 933.3 and 953.3 eV with the corresponding satellite peaks at 942.5 and 963.8 eV confirmed the Cu 2p core level in the +2 oxidation state (Figure 9) of CuO. In contrast, in the case of freshly reduced Cu@Mg/γ-Al_2O_3, the binding energies were observed at 932.6 and 952.3 eV corresponding to the zero oxidation state of copper in the reduced catalyst and no satellite peaks at the corresponding positions were observed which strongly suggest the complete reduction of small amount of CuO present in CuO@Mg/γ-Al_2O_3 to Cu(0)@Mg/γ-Al_2O_3 (Figure 9 and 10). The formation of Cu(0) nanoparticles by reduction was further confirmed with auger electron microscope (AES) analysis. Cu@Mg/γ-Al_2O_3 (fresh) and reused Cu@Mg/γ-Al_2O_3 (recovered after 5th run) were characterized by Auger electron microscopic analysis to confirm the zero oxidation state of the copper and no oxidation of metallic copper of Cu(0)@Mg/γ-Al_2O_3 was found even after successive runs. The kinetic energy for fresh and reused catalyst was found to be 919 eV (figure 11).

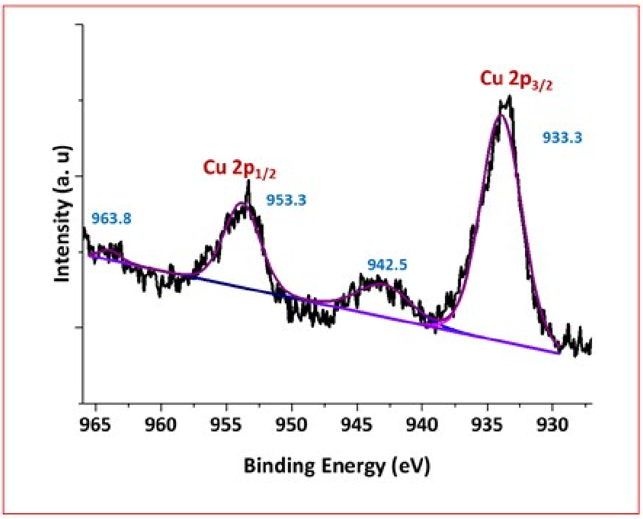

Figure 9. XPS spectrum of Cu@Mg/γ-Al_2O_3.

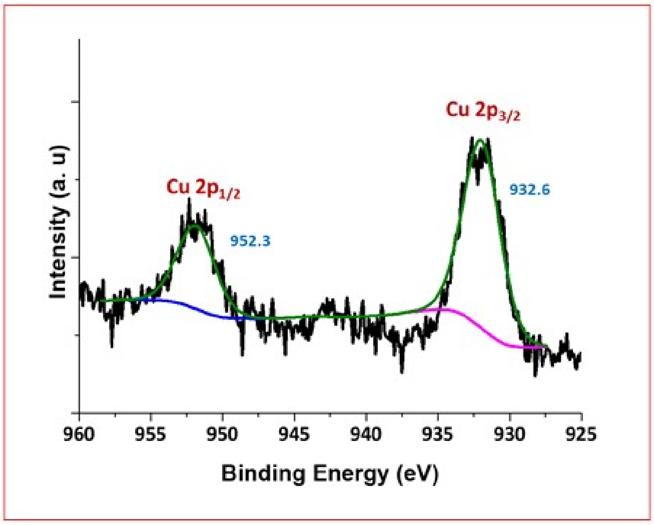

Figure 10. XPS spectrum of freshly reduced Cu@Mg/γ-Al_2O_3.

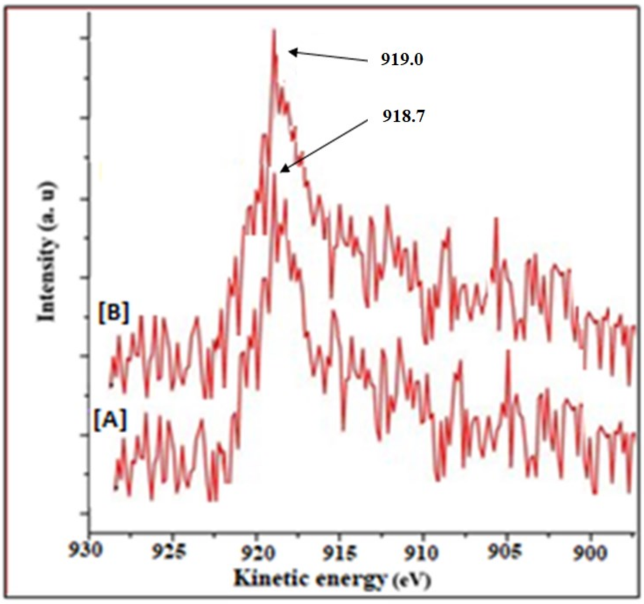

Figure 11. AES characterization of Cu@Mg/γ-Al_2O_3 [A] Fresh catalyst Cu@Mg/γ-Al_2O_3 and [B] Catalyst after 6th reuse.

Table 7. Effect of Cu/Mg on conversion of furfural to furfuryl alcohol.[a]

Sr. No	Cu/Mg	Furfural Conv.(%)	Furfuryl alcohol Yield (%)
1	4	99	90
2	5	99	94
3	10	99	80
4	15	99	65

[a]Reaction conditions: Furfural (2.6 mol%); catalyst Cu@Mg/γ-Al_2O_3 (0.1 g; Cu/Mg = X; where X= 5,10,15 and 4 on γ-Al_2O_3); H_2 (2 MPa); Temp (443.15 K); Time 5h.

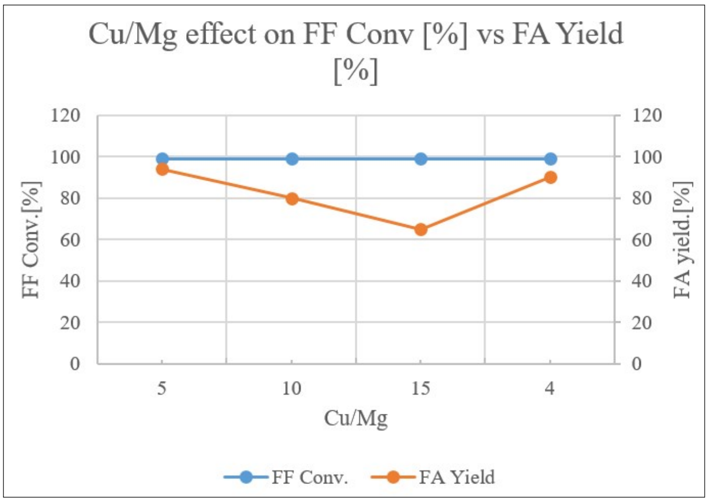

Figure 12. Effect of Cu/Mg on conversion of furfural to furfuryl alcohol.

Table 8. Comparison of catalyst efficiencies with the present system

Sr. No	Catalyst	Solvent	Temp (°C)	Pressure (bar)	FF Conv (%)	FA Selec (%)	Ref.
1	Ni−Mo−B/Al_2O_3	Methanol	80	50	99	90	9
2	Pt/C	Butanol	175	8	99	50	10
3	Pd/SiO2	Octane	230	1	69	10	11
4	Pt-Fe/MWNT	Ethanol	100	30	95.2	91.8	12
5	Pd−Cu/MgO	Water	110	6	100	99	13
6	Cu/SiO_2	Isopropanol	230	10	69	68	4
7	Cu/Al_2O_3	Water	90	2	81	81	5
8	Co-Cu/SBA-15	Isopropanol	170	20	99	80	6
9	Cu−Fe oxides	Octane	200	60	87	84	7
10	CuNiMgAl oxides	Ethanol	200	10	93	83	8
11	Cu@Mg/γ-Al_2O_3	-------	170	20	100	94	Present work

Table 9. Comparison of Cu@Mg/γ-Al_2O_3 activity with hydrotalcite derived copper catalysts in furfural hydrogenation

S. No	Catalyst	Cu (Wt %)	Mg (Wt%)	Temp (K)	P (bar)	Time (h)	FF Conv. (%)	Yield (%) FA	2-MF	THFA	Ref.
1	Cu-Mg-Al	17.9	>28	383	10	6	100	100	-	-	14
2[a]	Cu-Mg-Al	40	>12	423	-	6	100	100	-	-	15
3	Cu_2Al	82	18	513	10	1.5	>99	54.7	4.7	-	16
4	Cu_3Al	87	13	513	10	1.5	>99	44.9	3.7	-	16
5	$CuMgAlO_y$	11.2	>30	573	10	2	~50	~90	□10	-	17
6	Cu-Fe	64	-	433	90	5	91	90	0.5	-	18
7	Cu-Ni-Mg-Al	11.2	34	573	10	2	93.2	89.2	4.3	-	19
8	Cu@Mg/ γ -Al_2O_3	0.78	0.4	443	20	5	>99	94	2	4	Present work

3f. XRD analysis of synthesized catalysts:

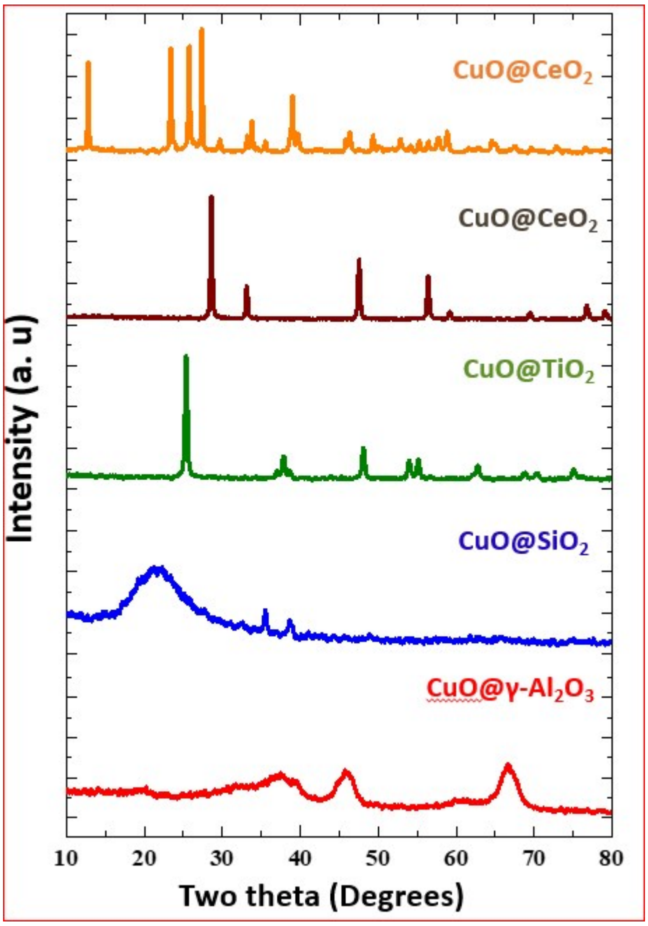

Figure 13. XRD patterns of calcined CuO supported on various supports

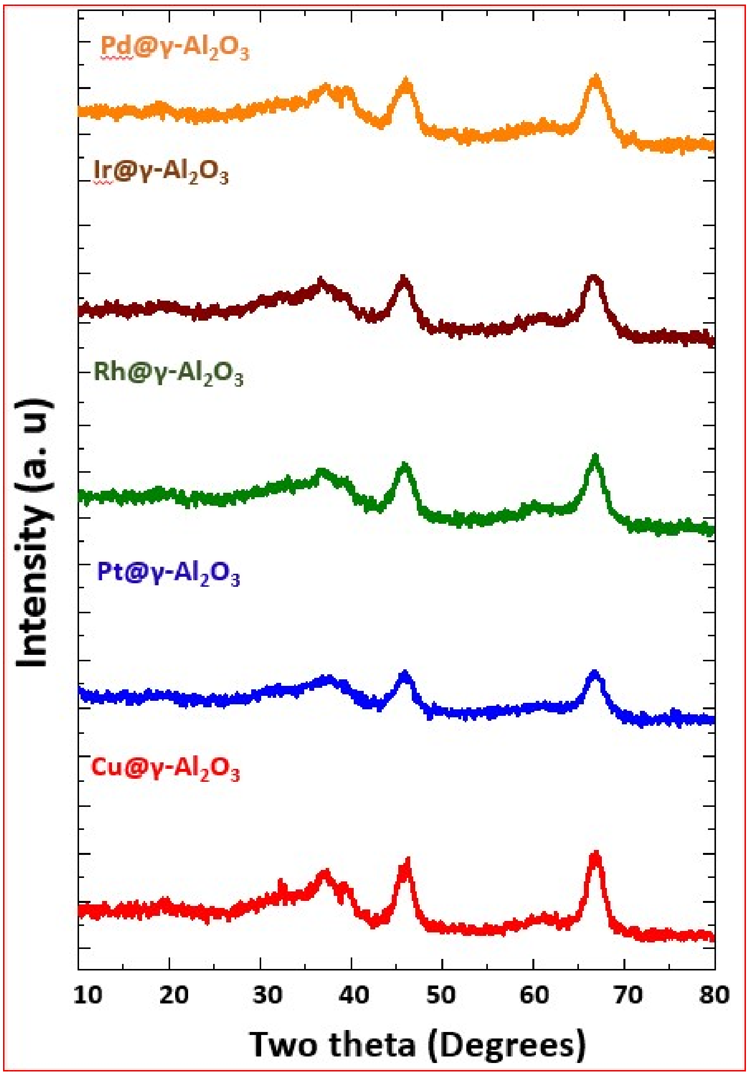

Figure 14. XRD patterns of Cu and various noble metals catalysts supported on Gamma alumina

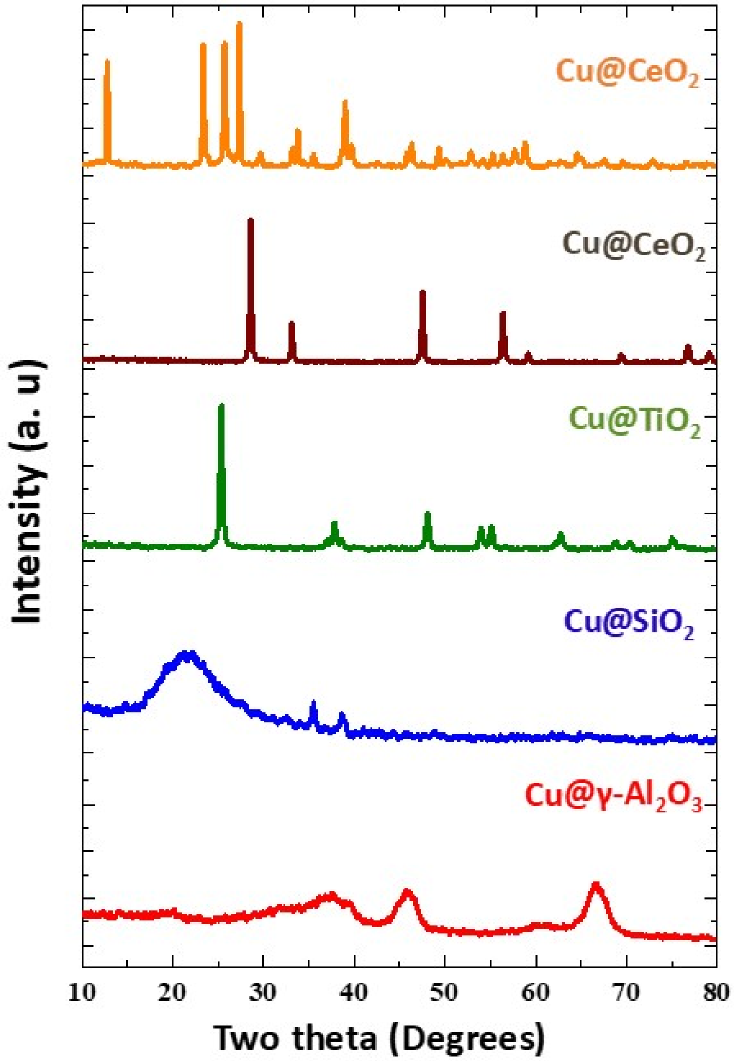

Figure 15. XRD patterns of reduced Copper catalysts supported on various supports

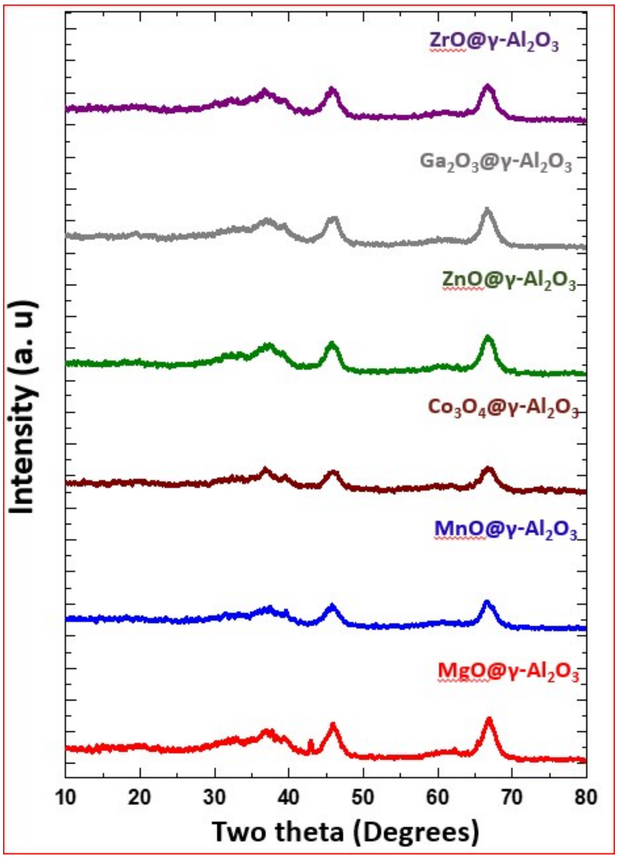

Figure 16. XRD patterns of promoter oxide on γ-Al_2O_3

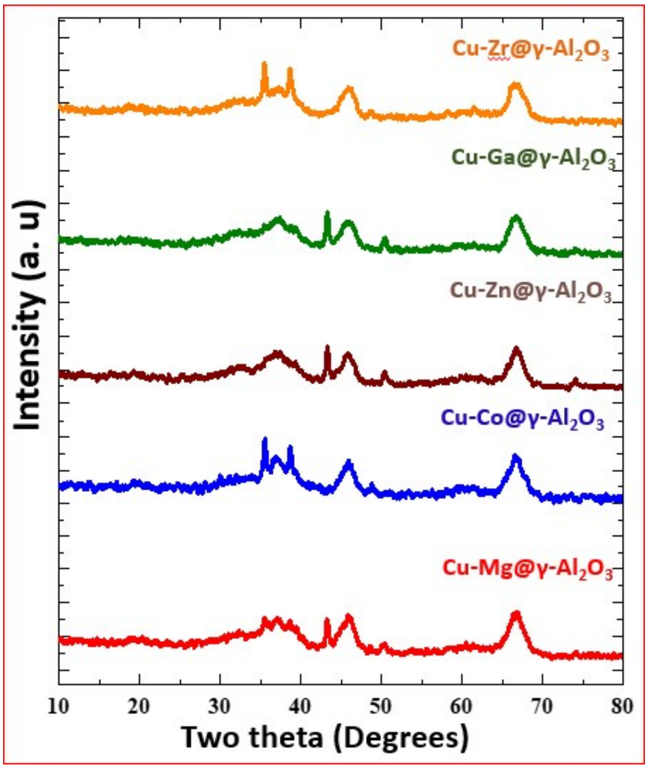

Figure 17. XRD patterns of Cu-catalyst supported on γ-Al_2O_3 incorporated with various metal oxidewith

4.0 General Procedure for the Hydrogenation Reaction:

Furfural (5.2 mol%), Cu@Mg/γ-Al_2O_3 (0.2 g), were taken in a 600-mL, round-PARR reactor equipped with an overhead stirrer and stirred for an appropriate time. The progress of the reaction was monitored by TLC and on completion of the reaction, the reaction mixture was centrifuged to separate the catalyst, the solid residue was washed with EtOAc (1 X 10 mL) to make the catalyst free from organic matter, the reaction mixture was diluted with water (20 mL), and then extracted with EtOAc (3 X 10 mL). The combined organic layers were washed with brine (10 mL) and dried over anhydrous Na_2SO_4. The solvent was evaporated under reduced pressure to yield the crude product. It was then purified by flash chromatography over silica gel (60–120 mesh) column using hexane/ethyl acetate (80:20) v/v as an eluent to afford the pure product. The products were characterized by ^{1}H NMR, ^{13}C NMR and mass spectrometric analysis.

5.0 Reaction Kinetics for the Hydrogenation Reaction:

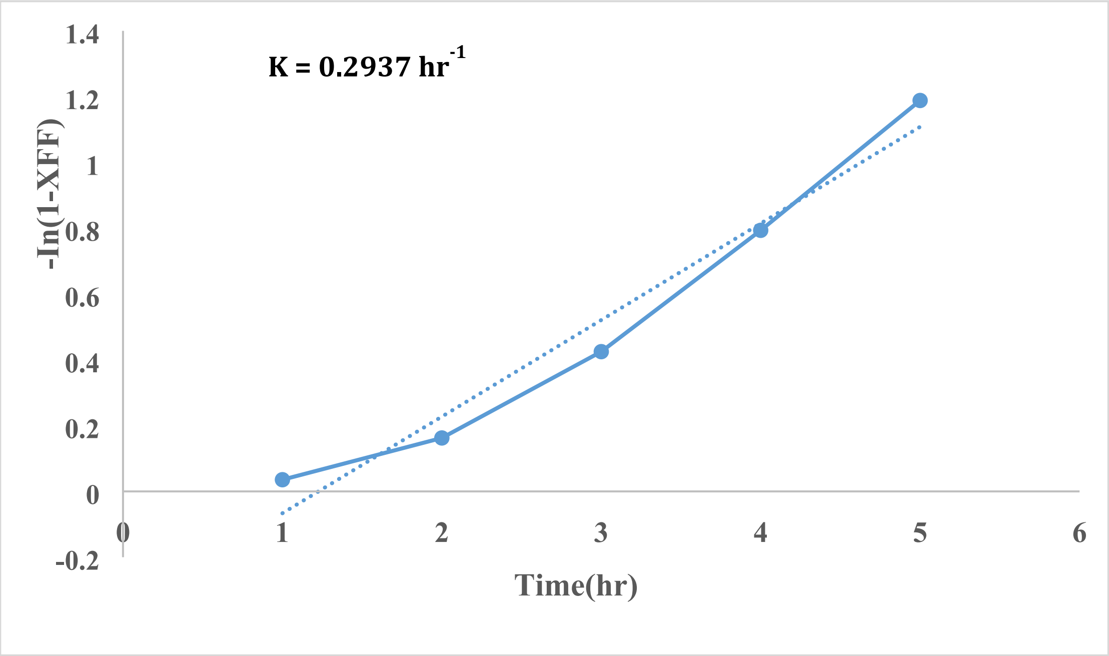

Figure 18. Conversion of furfural vs time for reaction rate constant estimation with Cu-Mg-Al- Hydrotalcite.

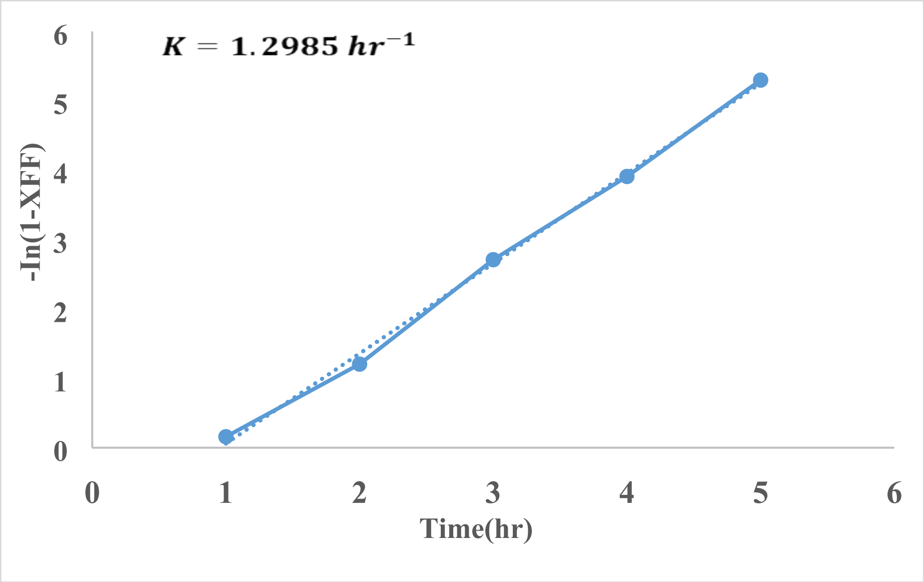

Figure 19. Conversion of furfural *vs* time for reaction rate constant estimation with Cu@Mg/γ -Al_2O_3

6.0 Inductively Coupled Plasma-Atomic Emission Analysis (ICP-AES): To test if any copper is leached during the reaction, ICP-AES, Inductively Coupled Plasma-Atomic Emission Spectroscopy analysis of the filtrates obtained after solid catalyst separation from reaction mixture to encounter leached metal in the filtrate was conducted. The Cu@Mg/γ-Al_2O_3 catalyst was separated from the reaction mixture by centrifugation, washed with ethyl acetate to make the catalyst free from organic matter, and finally with acetone, dried, and used in the next cycle. Almost consistent activity was noticed even after the fifth cycle. Copper content (0.2 mol%) of the fresh and used (after 5th cycle) catalyst was found to be almost the same (by ICP-AES). To check the heterogeneity of the catalyst, the hydrogenation of furfural was terminated at 16% conversion (after 30 min) and the catalyst was separated by simple centrifugation. The reaction was continued for an additional 4.5 hours and the conversion remained almost unchanged. Moreover, the filtrate was tested for copper by ICP-AES after the 6th cycle and no copper leaching was found for the five consecutive cycles. These studies clearly demonstrate that no leaching of copper from the catalyst was taken place during the reaction. .

Table 10. Leaching test of catalyst Cu@Mg/γ-Al_2O_3.

S. No	Catalyst	H_2 [MPa]	Time [h]	FF Conv.[%]	Yield [%] FA	2-MF	Furan	THF	THFA
1[a]	Cu@Mg/γ-Al_2O_3	2	0.5	16	85	0	3	trace	0
2[b]	Cu@Mg/γ-Al_2O_3	2	4.5	>99	94	2	4	0	0
3[c]	Cu@Mg/γ-Al_2O_3	2	5	>99	94	2	4	0	0

[a] **Reaction conditions**: FF (2.6 mol%), Catalyst (2 g, Cu-Mg: Cu@Mg; Cu 0.2 mol%, Mg 0.05 mol%), 443.15 K. [b] Reaction continued devoid of catalyst (catalyst removed by filtration). [c] Reaction with fresh catalyst .

Table 11. ICP-AES analysis of catalyst Cu@Mg/γ-Al_2O_3.

S. No	Catalyst	ICP-AES (Cu & Mg mol%)
1[a]	Cu@Mg/γ-Al_2O_3	Cu: 0.2001 & Mg: 0.0514
2[b]	Reaction filtrate	Cu: 0.0001 & Mg: trace
3[c]	Cu@Mg/γ-Al_2O_3	Cu: 0.2000 & Mg: 0.0511

[a] Fresh catalyst, [b] Reaction filtrate (catalyst removed by filtration), [c] Reaction with fresh catalyst .

7.0. ^{1}H and ^{13}C NMR spectrum of Furfuryl alcohol:

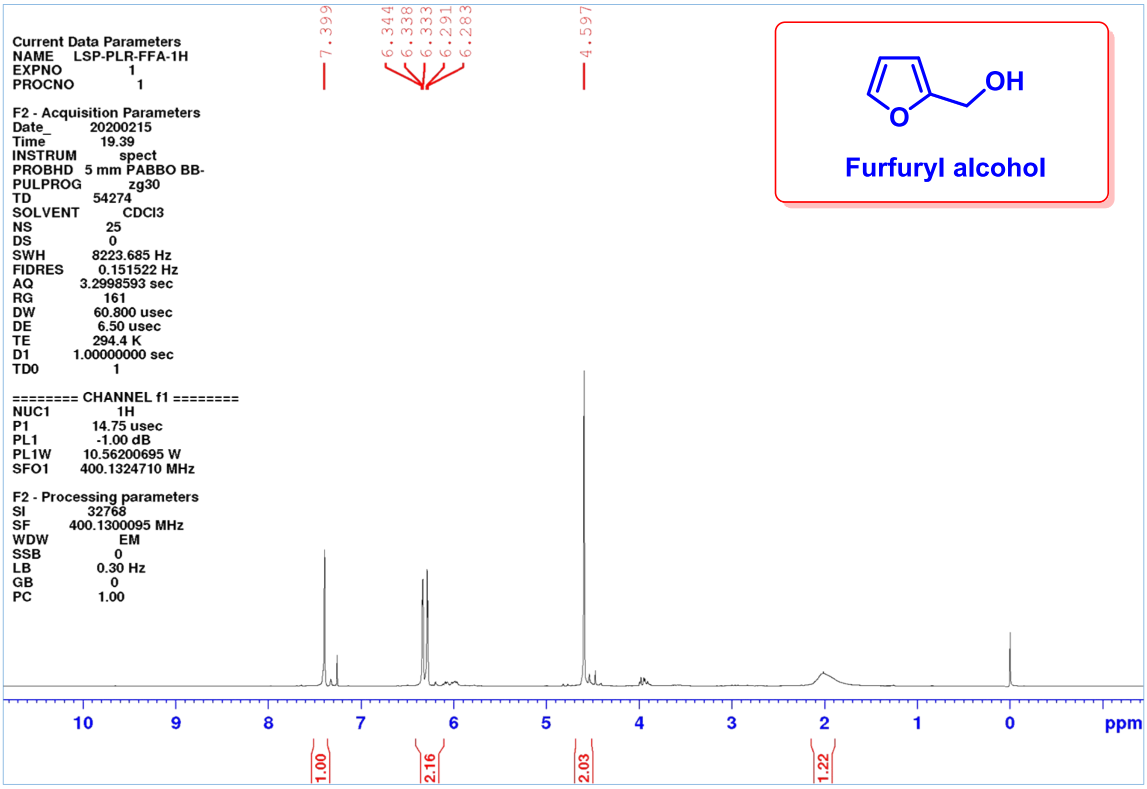

Figure 20. ^{1}H NMR spectra of furfuryl alcohol

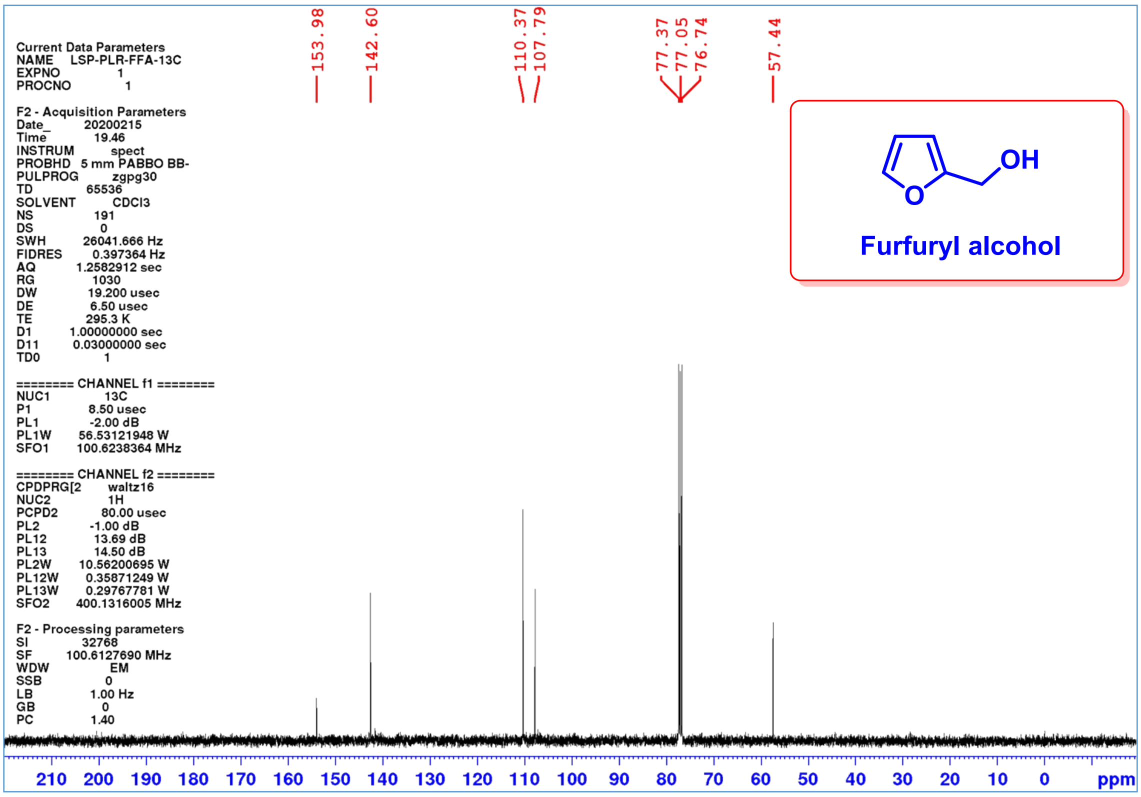

Figure 21. ^{13}C NMR spectra of furfuryl alcohol

8.0 References:

1. Y. Tang, Y. Liu, P. Zhu, Q. Xue, L. Chen and Y. Lu, *AIChE J.*, 2009, **55**, 1217-1228.
2. M. Su, R. Yang and M. Li, *Fuel*, 2013, **103**, 398-407.
3. F. W. Chang, W. Y. Kuo, K. C. Lee, *Appl. Catal.* 2003, **246**, 253-264.
4. S. Sitthisa, D. E. Resasco, *Catal. Lett.* 2011, **141,** 784-791
5. (*a*) M. Lesiak, M. Binczarski, S. Karski, W. Maniukiewicz, J. Rogowski, E. Szubiakiewicz, J. Berlowska, P. Dziugan, I. Witońska, *J. Mol. Catal. A: Chem.*, 2014, **395**, 337-348; (*b*) K. Fulajtárova, T. Soták, M. Hronec, I. Vávra, E. Dobročka, M. Omastová, Appl. *Catal. A.*, 2015, **502**, 78-85.
6. M. Audemar, C. Ciotonea, V. De Oliveira, S. Royer, A. Ungureanu, B. Dragoi, E. Dumitriu, F. Jérôme, *ChemSusChem.*, 2015, **8**, 1885-1891.
7. K. Yan, A. Chen, *Fuel.*, 2014, **115**, 101-108.
8. (*a*) B. M. Nagaraja, A. H. Padmasri, B. D. Raju and K. S. Rao. *J. Mol. Catal. A: Chem.*, 2007, **265**, 90-97. (*b*) B. M. Nagaraja, V. S. Kumar, V. Shasikala, A. H. Padmasri, B. Sreedhar, B. D. Raju and K. S. Rao, *Catal Commun*, 2003, **4,** 287-293.

9. S. Wei, H. Cui, J. Wang, S. Zhuo, W. Yi, L. Wang, Z. Li, *Particuology.*, 2011, **9**, 69-74.
10. M. J. Taylor, L. J. Durndell, M. A. Isaacs, C. M. Parlett, K. Wilson, A. F. Lee, G. Kyriakou, *Appl. Catal. B.*, 2016, **180**, 580-585.
11. S. Sitthisa and D. E. Resasco. *Catal. Lett.*, 2011, **141**, 784-791.
12. L. Liu, H. Lou, M. Chen, *Appl. Catal. A.*, 2018, **550**, 1-10.
13. K. Fulajtárova, T. Soták, M. Hronec, I. Vávra, E. Dobročka, M. Omastová, *Appl. Catal. A.*, 2015, **502**, 78-85.
14. M. M. Villaverde, N. M. Bertero, T. F. Garetto, A. J. Marchi, *J. Catal. Today.,* 2013, **213**, 87-92.
15. M. M. Villaverde, T. F. Garetto, A. J. Marchi, *Catal. Commun.*, 2015, **58**, 6-10.
16. J. Zhang and J. Chen, *ACS Sustainable Chem. Eng.,* 2017, **5**, 5982-5993.
17. H. Liangke, X. Chenghua, L. Wenbiao, L. Jianying, L. Jun, D. Xingjie, G. Yan, Y. Yingchun., *Chin. J. of Catal.,* 2010, **31**, 461-465.
18. K. Yan, J. Y. Liao, X. Wu, X. M. Xie. *Rsc Adv.*, 2013, **3**, 3853-3856.
19. C. Xu, L. Zheng, D. Deng, J. Liu, S. Liu. *Catal Commun*, 2011, **12**, 996-999.
